# Phosphodiesterase 4 inhibition after retrieval switches the memory fate favoring extinction instead of reconsolidation

**DOI:** 10.1038/s41598-023-47717-1

**Published:** 2023-11-21

**Authors:** Jeferson Machado Batista Sohn, Nathalie Carla Cardoso, Ana Maria Raymundi, Jos Prickaerts, Cristina Aparecida Jark Stern

**Affiliations:** 1 Department of Pharmacology, Federal University of Parana, Curitiba, PR Brazil; 2 Department of Psychiatry and Neuropsychology, School of Mental Health and Neuroscience, University of Maastricht, Maastricht, The Netherlands

**Keywords:** Emotion, Learning and memory, Molecular neuroscience

## Abstract

Phosphodiesterase 4 (PDE4), an enzyme expressed in the dorsal hippocampus (DH), hydrolyzes the cAMP, limiting the PKA-induced CREB phosphorylation (pCREB) and BDNF expression. Depending on the brain region, PKA and pCREB mediate reconsolidation or extinction, whereas BDNF is mainly related to extinction facilitation. The mechanisms underpinning the switch between reconsolidation and extinction are relatively unknown. Here, we tested the hypothesis that PDE4 might control these processes. We showed in Wistar rats submitted to contextual fear conditioning that PDE4 inhibition with roflumilast (ROF) within the DH, after a short retrieval, did not change freezing behavior after one day (TestA_1_). After 10 days, the ROF-treated group significantly reduced the expression of freezing behavior. This effect depended on retrieval, Test A_1_ exposure, and reinstated after a remainder foot shock, suggesting an extinction facilitation. The ROF effect depended on PKA after retrieval or, protein synthesis after Test A_1_. After retrieval, ROF treatment did not change the pCREB/CREB ratio in the DH. It enhanced proBDNF expression without changing pre-proBDNF or mature BDNF in the DH after Test A_1_. The results suggest that the inhibition of PDE4 in the DH after a short retrieval changes the memory sensibility from reconsolidation to extinction via regulating proBDNF expression.

## Introduction

Memory retrieval is a dynamic process whereby a fear memory may enter into a labile state and undergo reconsolidation, otherwise, extinction can take place^1,2^. Prolonged or repeated retrieval sessions trigger extinction, generating an inhibitory learning that transiently impairs the original fear memory expression^1–3^. Short retrieval sessions may induce reconsolidation. Disrupting reconsolidation has been associated with a permanent reduction of fear expression because it changes the original fear memory^4,5^. Although both phenomena depend on retrieval, the mechanism underpinning the switch between reconsolidation and extinction is relatively unknown. Advancing this knowledge is relevant to reducing the impact of maladaptive memories that are associated with psychiatric disorders such as post-traumatic stress disorder (PTSD)^1^.

Using an inhibitor of phosphodiesterase 4 (PDE4) after retrieval of an inhibitory avoidance task, it was suggested that PDE4 is involved in the switch from extinction to reconsolidation^6^. The activity of PDE4 is regulated by protein kinase A (PKA) phosphorylation^7^. When activated, PDE4 hydrolyzes the cAMP, interrupting the cAMP/PKA signaling pathway^7,8^. PDE4 is highly expressed in the dorsal hippocampus (DH), a brain area involved either in reconsolidation and extinction of contextual fear memory^6,9–11^. However, it remains unclear whether PDE4 activity in the DH contributes to fear memory reconsolidation.

A classical downstream pathway induced by cAMP/PKA is CREB phosphorylation (pCREB) and enhancement of BDNF expression^8,12,13^. BDNF is involved in fear memory consolidation and extinction^3,14–16^. Specifically, whereas the BDNF mature portion underlies fear maintenance, the proBDNF portion is related to extinction facilitation^16^. By interacting with the TrkB receptor, BDNF increases PKMζ activity and memory persistence^17^. Of note, PKMζ is involved in the maintenance of long-term fear memory^18,19^. Considering the lack evidence on the role of PDE4 and the PKA/CREB/BDNF pathway on reconsolidation and the transition between reconsolidation and extinction of contextual fear memory, we sought to examine how PDE4 inhibition in the DH with roflumilast (ROF; a selective PDE4 inhibitor), 5 min after a short fear retrieval, would control reconsolidation and/or the transition from reconsolidation to extinction. Then, the animals underwent contextual fear conditioning. The effects of PDE4 inhibition in the DH, or i.p., were evaluated through behavioral, pharmacological, and immunoblotting approaches. Animals were exposed to a short retrieval session and immediately after they received the treatment with ROF. The effects of ROF were tested in subsequent behavioral tests conducted 1 and 10 days after treatment. The involvement of PKA and protein synthesis in the ROF-induced effects were also evaluated, as well as the expression of CREB/pCREB, BDNF fractions and PKMζ in the DH.

## Results

### PDE4 inhibition in the DH after retrieval reduced freezing behavior in Test A_2_ but not in Test A_1_

**Figure 1:**
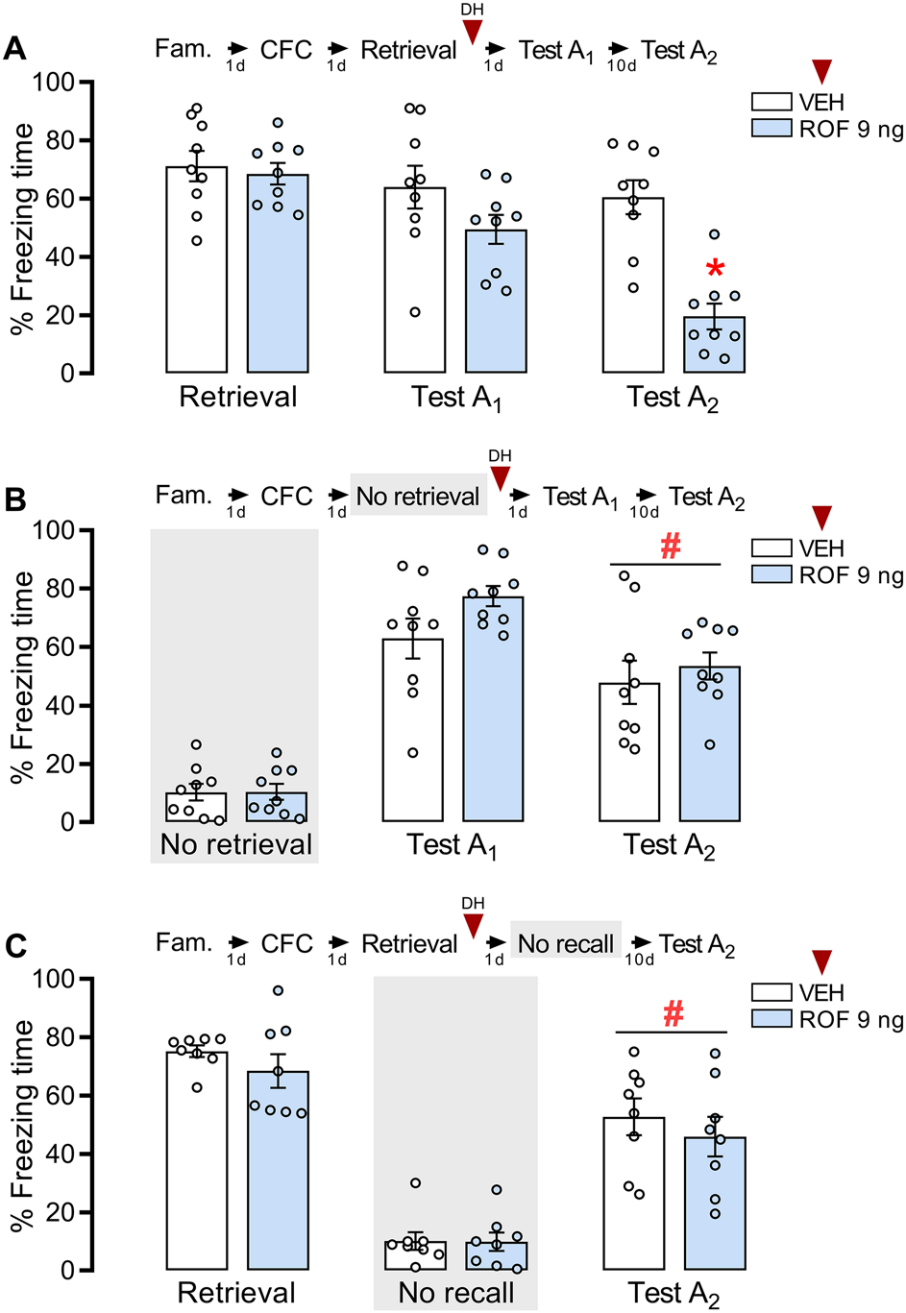
Effects of PDE4 inhibition in the DH after a short retrieval session. The experimental design is represented above the graphs. The red arrows represent the moment of treatment. (**A**) PDE4 inhibition after retrieval did not induce any effects during Test A_1_. However, animals that were treated with ROF presented less freezing behavior than controls in Test A_2_. n: ROF = 9; VEH = 9. (**B**) The omission of the retrieval session abolished the effect of ROF in Test A_2_. n: ROF = 9; VEH = 9. (**C**) The omission of Test A_1_ 24 h after the retrieval and treatments, abolished the effect of ROF in Test A_2_. n: ROF = 8; VEH = 8. The data is represented by mean ± S.E.M. and the individual values of the percentage of freezing expressed by animals during each session. The * represents a significant difference (*P < 0.05) compared to VEH in the same session. The # represents a significant difference (#P < 0.05) comparing the groups to themselves at previous Context A exposure.

To evaluate the effects of PDE4 inhibition on reconsolidation, fear-conditioned animals received ROF (selective PDE4 inhibitor, 9 ng/0.5 µL/side) or VEH into the DH 5 min after retrieval (n = 9/group). Repeated-measures ANOVA showed a significant interaction between context re-exposure and treatment (F_2,32_ = 10.86; *P* < 0.001; η^2^ = 0.40). Figure 1A shows that ROF-treated animals presented less freezing behavior than controls during Test A_2_ (*P* < 0.01). No significant differences between groups during retrieval (*P* = 0.73) and Test A_1_ (*P* = 0.15) were observed, suggesting that the inhibition of PDE4 activity in the DH impairs the fear memory sustaining over time. No significant effects were observed in memory generalization (Table S1).

### The effects of PDE4 inhibition in the DH depend on memory retrieval and Test A_1_ exposure

To evaluate whether PDE4 inhibition effects depend on memory retrieval, fear-conditioned animals received ROF or VEH into the DH (n = 9/group) 5 min after exposure to the no-retrieval session (exposure to the unpaired Context B). Student’s t-test showed no difference between groups during Context B exposure (t_16_ = 0.031; *P* = 0.97). Repeated-measures ANOVA showed no significant interaction of Context A re-exposure and treatment (F_2,32_ = 1.34; *P* = 0.265; η^2^ = 0.08) nor significant treatment effect (F_1,16_ = 1.93; *P* = 0.183; η^2^ = 0.11), suggesting that the ROF effect depends on memory retrieval. A significant difference in Context A re-exposure was observed (F_2,32_ = 25.58; *P* < 0.001; η^2^ = 0.61). Figure 1B shows that during Test A_2_ both groups reduced freezing time compared to Test A_1_.

To evaluate whether the effects of PDE4 inhibition depend on Test A_1_ exposure 24 h after treatment, fear-conditioned animals received ROF or VEH into the DH 5 min after retrieval (n = 8/group) and in the next day, were exposed to Context B for 3 min. Repeated-measures ANOVA showed no significant interaction between Context A re-exposure and treatment (F_2,14_ = 0.00008; *P* = 0.993; η^2^ < 0.01), nor a significant treatment effect (F_1,14_ = 0.97; *P* = 0.341; η^2^ = 0.06), suggesting that the ROF effect depends on Test A_1_ exposure. A significant effect of Context A re-exposure (F_2,14_ = 35.07; *P* < 0.001; η^2^ = 0.71) was observed. Student’s t-test showed no difference between groups during Context B exposure (t_14_ = − 0.06; *P* = 0.950). Figure 1C shows that during Test A_2_ both groups reduced freezing behavior compared to the retrieval session.

### The reinstatement test spared the effect of PDE4 inhibition in the DH

**Figure 2:**
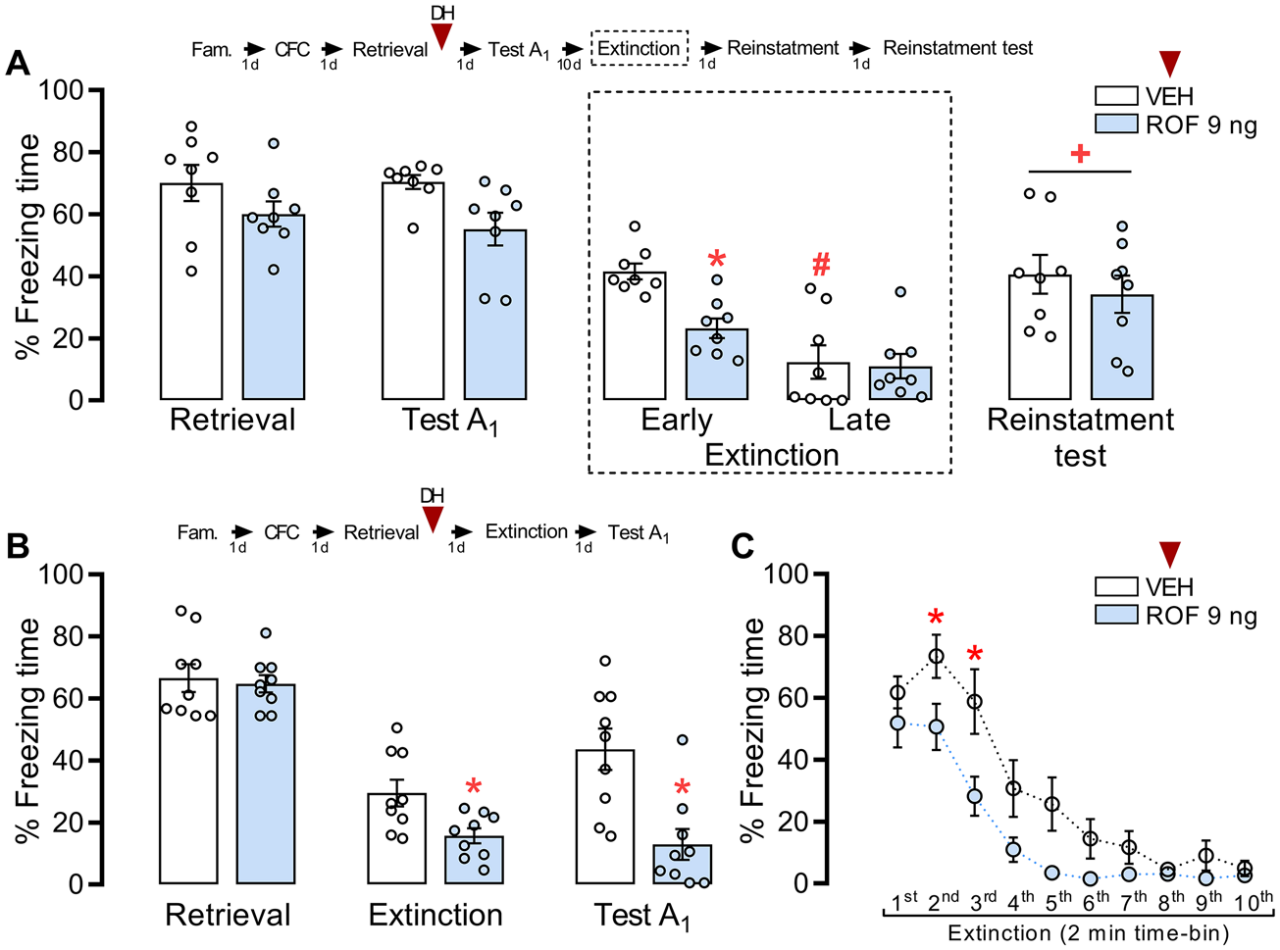
Effects of PDE4 inhibition on fear memory reinstatement and fear memory extinction. The experimental design is represented above the graphs. The red arrows represent the moment of treatment. (**A**) No differences were observed among groups during retrieval and Test A_1_. However, animals treated with ROF presented less freezing behavior, 10 days after Test A_1_, during early extinction (first 3 min) compared to control in the same session. In late extinction (last 3 min), the control group showed less freezing behavior compared to itself during early extinction. After a reminder shock, in the reinstatement test, both groups showed higher freezing levels than themselves during late extinction. (**B**) Animals treated with ROF after retrieval presented less freezing behavior than controls in the extinction session, suggesting facilitation of fear extinction. 24 h after extinction, in Test A_1_, the ROF-treated group presented less freezing than controls. (**C**) The extinction session separated into 2 min time-bin showed that ROF-treated animals reduced the fear expression faster than controls. The data in (**A**) and (**B**) is represented by mean ± S.E.M. and the individual values of the percentage of freezing expressed by animals during each session. The data in (**C**) is represented by mean ± S.E.M. expressed in extinction divided into 2 min time-bin. The * represents a significant difference (*P < 0.05) compared to VEH in the same session. The # represents a significant difference (#P < 0.05) compared to the same group during early extinction. The + represents a significant difference (+ P < 0.05) compared to the same group during late extinction. n: VEH = 8; ROF = 8.

To assess whether a reminder foot shock induces memory reinstatement, fear-conditioned animals received ROF or VEH into the DH 5 min after retrieval (n = 8/group). 10 days after Test A_1,_ the animals underwent fear extinction. After 24 h, animals received a mild foot shock in Context C. One day later, the reinstatement test in Context A was evaluated. Repeated-measures ANOVA showed no interaction between Context A re-exposure and treatment (F_4,56_ = 0.96; *P* = 0.435; η^2^ = 0.06), but a significant effect of treatment (F_1,14_ = 18.59; *P* < 0.001; η^2^ = 0.57) and of re-exposure (F_4,56_ = 41.75; *P* < 0.001; η^2^ = 0.75). According to our previous result, ROF-treated rats showed less freezing behavior during early extinction (first 3 min; Fig. 2A) than the controls (*P* = 0.04). The control group reduced the fear expression in late extinction (last 3 min) compared to early extinction (*P* < 0.01), indicating extinction formation. During the reinstatement test, both groups increased fear response compared to late extinction (*P* < 0.01). No difference was observed between the ROF and VEH groups in this session (*P* = 0.33), suggesting that PDE4 inhibition effects are associated with extinction facilitation.

### The inhibition of PDE4 in the DH after retrieval facilitates fear extinction

To further investigate that ROF effects depend on a shift of reconsolidation to extinction, fear-conditioned animals received ROF or VEH into the DH 5 min after retrieval (n = 9/group). After 24 h they underwent fear extinction and one day later, the animals were exposed to Test A_1._ Repeated-measures ANOVA showed a significant interaction between Context A re-exposure and treatment (F_2,32_ = 9.56; *P* < 0.001; η^2^ = 0.37). ROF-treated animals expressed less freezing than controls in total extinction and Test A_1_ (*P* < 0.04; Fig. 2B), but not during retrieval (*P* = 0.77). Repeated-measures ANOVA showed a significant interaction between the time-bin and treatment along the extinction session (F_9,144_ = 2.15; *P* = 0.029; η^2^ = 0.12). As shown in Fig. 2C, the ROF-treated animals presented a faster reduction in freezing time than controls during extinction (from 3rd to 4th time-bin; *P* < 0.04), suggesting that PDE4 inhibition after a short retrieval session facilitates the extinction process.

### Protein synthesis inhibition in the DH after Test A_1_ spared the effect of PDE4 inhibition after retrieval

**Figure 3:**
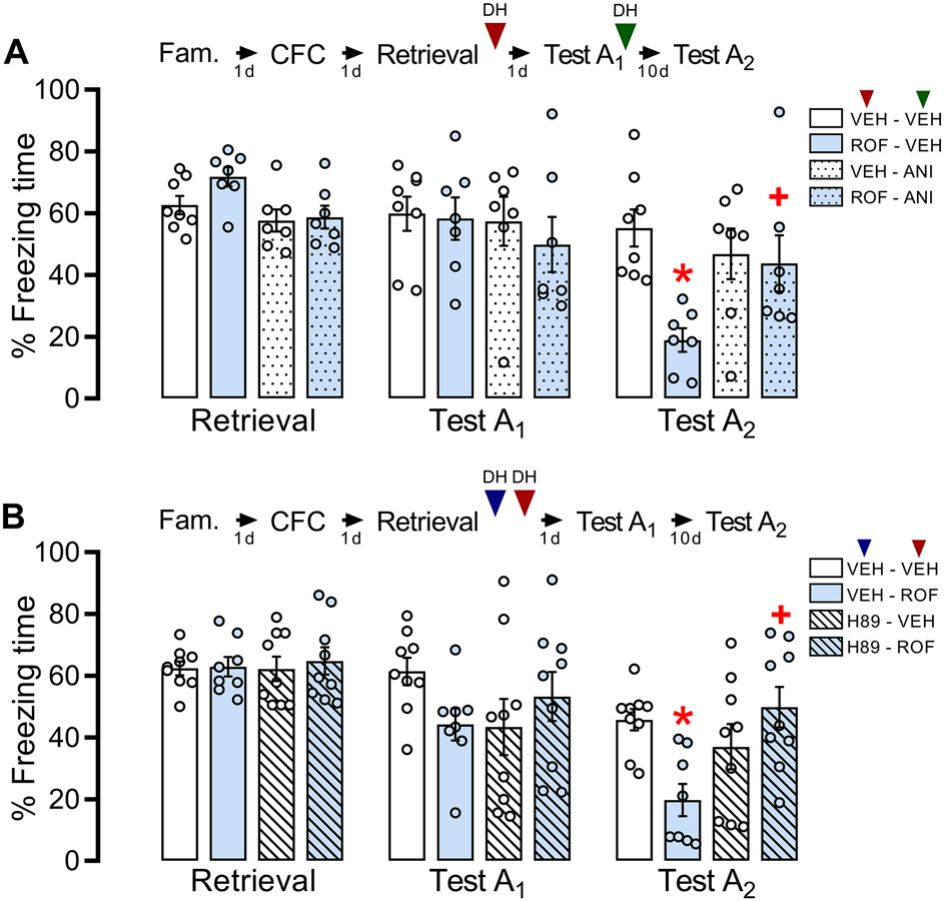
Effects of protein synthesis or PKA inhibition on the effects induced by PDE4 inhibition in the DH after a short retrieval session. The experimental design is represented above the graphs. The red arrows represent the moment of treatment with ROF or VEH. The green arrow represents the moment of treatment with ANI or its VEH. The blue arrow represents the moment of treatment with H89 or its VEH. (**A**) No differences were observed among groups during retrieval and Test A_1_. However, animals that received ROF-VEH presented less freezing behavior during Test A_2_ when compared to the control (VEH-VEH) in the same session. Moreover, the group that received ROF-ANI, presented higher freezing behavior than the group that received ROF-VEH. n: VEH-VEH = 8; ROF-VEH = 7; ROF-ANI = 7; VEH-ANI = 7. (**B**) No differences were observed among groups during retrieval and Test A_1_. However, the animals treated with ROF-VEH presented less freezing behavior during Test A_2_ compared to the control (VEH-VEH) in the same session. Moreover, the group that received ROF-H89, presented higher freezing behavior than the group that received ROF-VEH. n: VEH-VEH = 9; ROF-VEH = 8; ROF-H89 = 9; VEH-H89 = 9. The data is represented by mean ± S.E.M. and the individual values of the percentage of freezing expressed by animals during each session. The * represents a significant difference (*P < 0.05) compared to the VEH-VEH group in the same session. The + represents a significant difference (+ P < 0.05) compared to the ROF-VEH group in the same session.

To evaluate whether protein synthesis induced by Test A_1_ underlies the effect of ROF, fear-conditioned animals received ROF or VEH into the DH 5 min after retrieval. On the next day, immediately after Test A_1_, each group received anisomycin (protein synthesis inhibitor; ANI) or VEH into the DH (n = 7–8/group). Two-way repeated-measures ANOVA showed significant interaction among Context A re-exposure, pretreatment, and treatment (F_2,56_ = 3.16; *P* = 0.050; η^2^ = 0.10). As observed in Fig. 3A, there are no differences among groups during retrieval or Test A_1_. During Test A_2_, the ROF-VEH group presented a significant reduction of freezing behavior compared to VEH-VEH (*P* < 0.01) and ROF-ANI (*P* = 0.03). This effect was spared in the ROF-ANI group when compared to the VEH-ANI (*P* = 0.11) and VEH-VEH (*P* = 0.99), suggesting that protein synthesis after Test A_1_ underlies the effects observed by PDE4 inhibition (Fig. 3).

### The effect of PDE4 inhibition after retrieval depends on PKA

To evaluate whether PKA activity is involved in ROF effects, fear-conditioned rats received immediately after retrieval a pretreatment with H89 (PKA inhibitor) or VEH and, 5 min later, each group received ROF or VEH into the DH (n = 8–9/group). Two-way repeated-measures ANOVA showed a significant interaction among Context A re-exposure, pretreatment, and treatment (F_2,62_ = 4.28; *P* = 0.018; η^2^ = 0.18). As shown in Fig. 3B, no differences were detected among groups during retrieval or Test A_1._ During Test A_2_ the ROF-VEH group presented a significant reduction in freezing behavior compared to controls (*P* = 0.01). This effect was spared in the H89-ROF group (*P* = 0.61), suggesting that PKA activity after retrieval underlies the ROF effects on Test A_2_.

### Systemic inhibition of PDE4 after retrieval reduced freezing behavior in Test A_2_ but not in Test A_1_

To investigate whether ROF systemic administration would produce similar effects to those observed within the DH, fear-conditioned rats received VEH or ROF 0.1 mg/kg (n = 9/groups) i.p. 5 min after memory retrieval. Repeated-measures ANOVA showed a significant interaction between treatment and Context A re-exposure (F_2,32_ = 4.30; *P* = 0.022; η^2^ = 0.21). As shown in Fig. S2A, ROF-treated animals showed less freezing behavior in Test A_2_ than VEH-treated (*P* < 0.01). No differences were detected during retrieval and Test A_1_. When Test A_1_ was omitted, repeated-measures ANOVA showed no significant interaction between treatment and Context A re-exposure (F_1,18_ = 3.17; *P* = 0.092; η^2^ = 0.15) nor treatment effect (F_1,18_ = 0.65; *P* = 0.43; η^2^ = 0.03) but, a significant effect in context A re-exposure (F_1,18_ = 8.88; *P* = 0.008; η^2^ = 0.33). As shown in Figure S2B, Test A_1_ omission abolished the ROF effects in Test A_2_, suggesting that systemic or intra-DH inhibition of PDE4 after retrieval reduces freezing behavior.

### PDE4 inhibition did not change total and phosphorylated CREB expression in the DH after retrieval or Test A_1_

To investigate the involvement of CREB, BDNF, and PKMζ in ROF-induced effects, fear-conditioned rats received VEH or ROF 0.1 mg/kg (n = 12–13/group), i.p., 5 min after retrieval and had their DH dissected 90 min after treatment or Test A_1._

**Figure 4:**
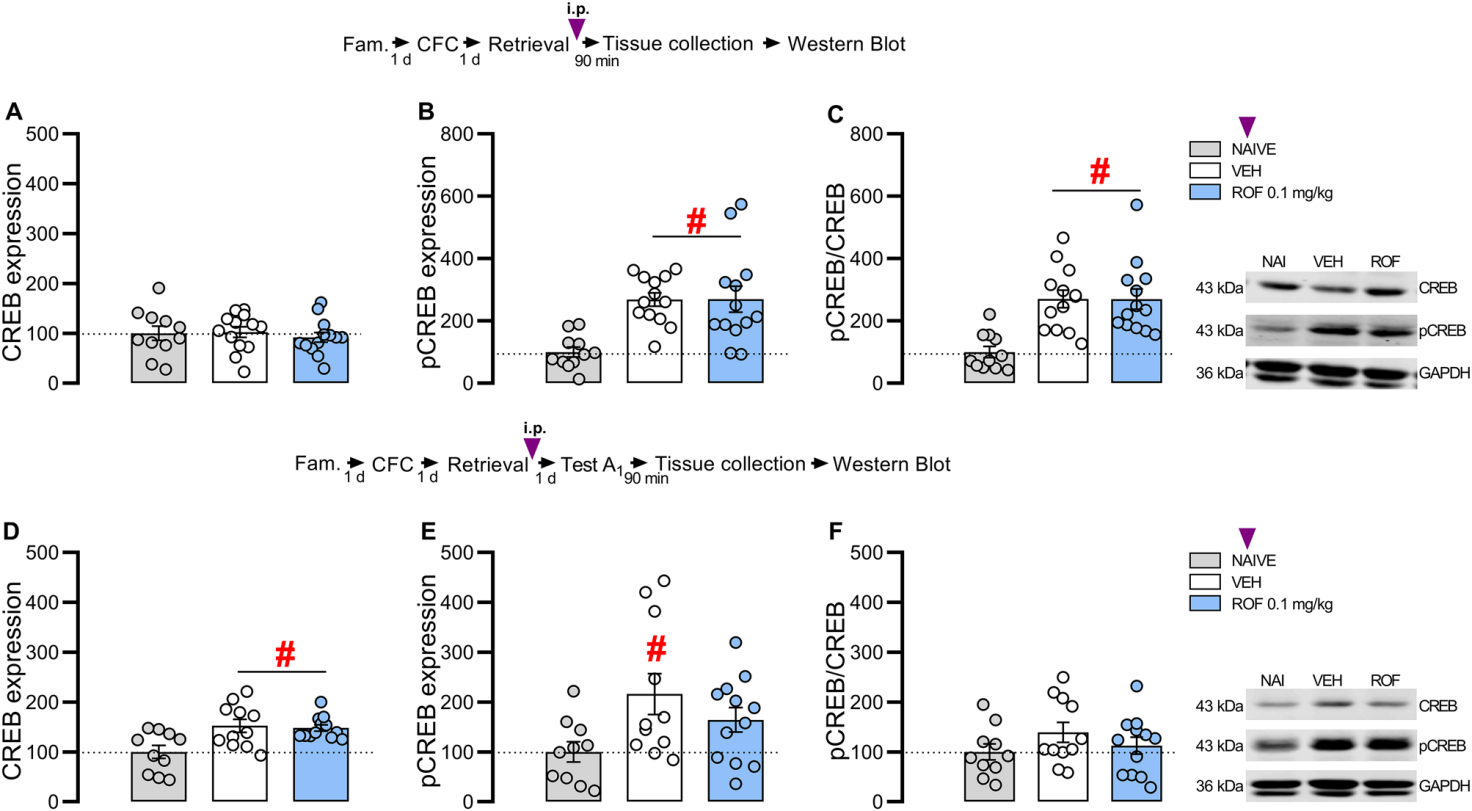
Effects of PDE4 inhibition on total and phosphorylated CREB expression in the DH. The experimental design is represented above the graphs. The purple arrows represent the moment of treatment with ROF or VEH. (**A**–**C**) represent the samples collected 90 min after retrieval and treatment. (**A**) No changes were observed in CREB expression among groups. (**B**) The VEH and ROF groups presented higher pCREB expression than naive. **C)** The VEH and ROF groups presented a higher pCREB/CREB ratio than naïve; (n: NAIVE = 11; VEH = 13; ROF = 13). (**D**–**F**) represent the samples collected 90 min after Test A_1_. (**D**) The VEH and ROF groups presented higher CREB expression than naive. (**E**) The VEH group showed higher pCREB expression than naive. No differences were observed between ROF and naive or VEH groups. (**F**) No differences were detected in the pCREB/CREB ratio among groups. (n: NAIVE = 10; VEH = 11; ROF = 12). The data is represented by mean ± S.E.M. and the individual values of the percentage (compared to the naive group) of CREB or pCREB expression normalized for GAPDH or pCREB/CREB ratio. The # represents significant differences (#P < 0.05) compared to the naive group.

After retrieval, one-way ANOVA showed no significant effect of treatment for CREB expression (F_2,34_ = 0.25; *P* = 0.783; η^2^ = 0.01; Fig. 4A). A significant treatment effect was detected for pCREB expression (F_2,34_ = 10.03; *P* < 0.001; η^2^ = 0.37) and the pCREB/CREB ratio (F_2,34_ = 11.82; *P* < 0.001; η^2^ = 0.41). As shown in Fig. 4B, C, an increase in pCREB expression and in the pCREB/CREB ratio was observed in the VEH and ROF 0.1 compared to the naive group (*P* < 0.01), suggesting a retrieval-induced enhancement of pCREB expression in the DH, without further enhancement after PDE4 inhibition.

After Test A_1_, one-way ANOVA showed significant effects of treatment for CREB (F_2,30_ = 6.82; *P* = 0.004; η^2^ = 0.31) and pCREB (F_2,30_ = 3.54; *P* = 0.042; η^2^ = 0.19). As observed in Fig. 4D, the VEH and ROF 0.1 groups presented higher CREB expression than the naive (*P* < 0.01), whereas only the VEH group presented a significant enhancement of pCREB compared to naive (*P* = 0.03; Fig. 4E). No significant differences were detected in the pCREB/CREB ratio (F_2,30_ = 1.24; *P* = 0.305; η^2^ = 0.08). As observed in Fig. 4F the groups presented a similar pCREB/CREB ratio.

### PDE4 inhibition increases proBDNF expression in the DH after Test A_1_

**Figure 5:**
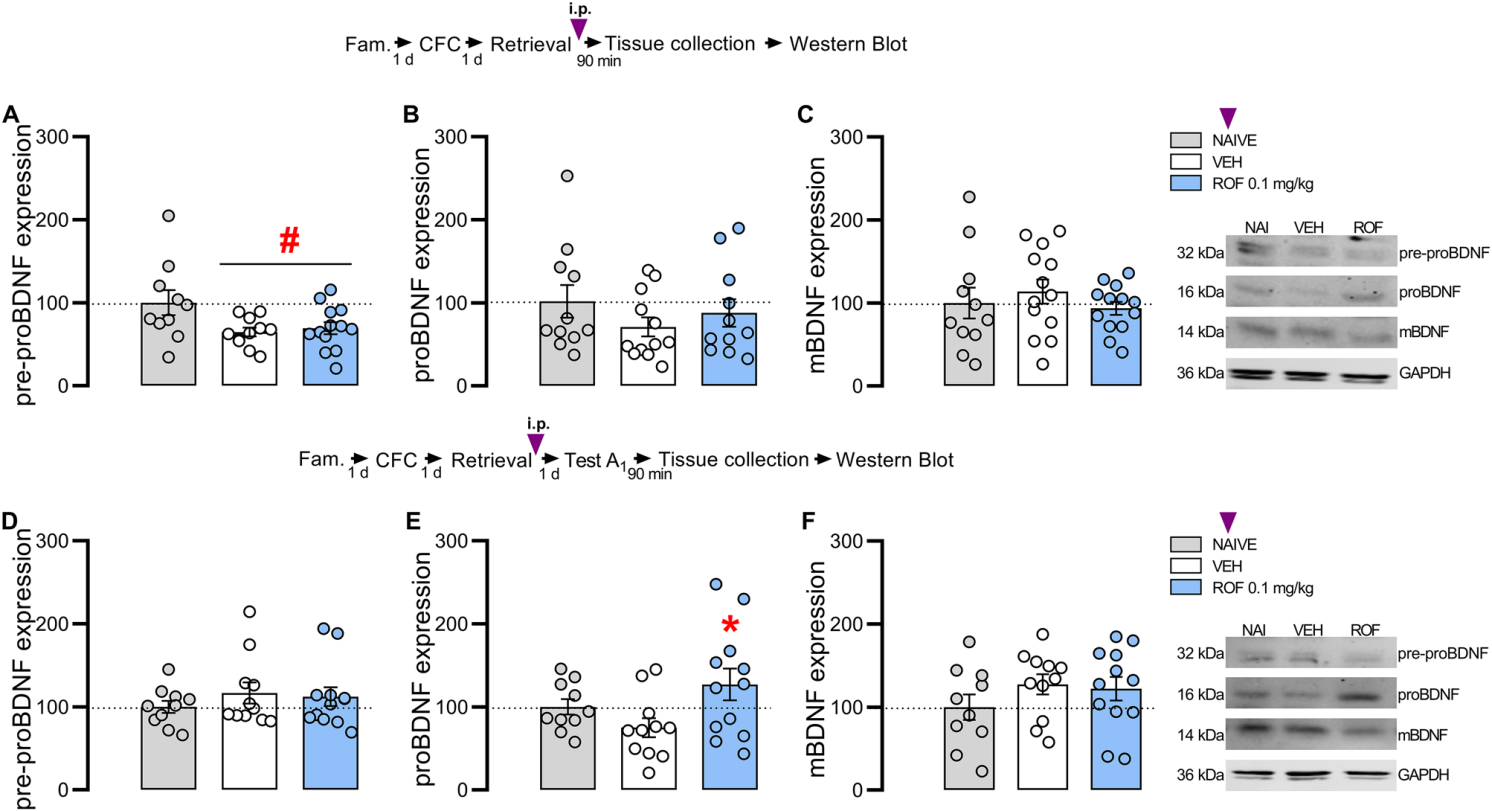
Effects of PDE4 inhibition on the BDNF expression in the DH. The experimental design is represented above the graphs. The purple arrows represent the moment of treatment with ROF or VEH. (**A**–**C**) Represent the samples collected 90 min after retrieval and treatment. (**A**) The VEH and ROF groups presented lower pre-proBDNF than the naive group. (**B**) No differences were detected in proBDNF among groups. (**C**) No differences were detected in mBDNF among groups; (n: NAIVE = 10–11; VEH = 11–13; ROF = 11–13). (**D**–**F**) Represent the samples collected 90 min after Test A_1_. (**D**) No differences were detected in pre-proBDNF among groups. (**E**) The ROF-treated group showed higher proBDNF expression than the VEH-treated group. (**F**) No differences were detected in mBDNF expression among groups; (n: NAIVE = 10; VEH = 11; ROF = 12). The data is represented by mean ± S.E.M. and the individual values of the percentage (compared to the naive group) of pre-proBDNF, proBDNF or mBDNF expression normalized for GAPDH. The * represents a significant difference (*P < 0.05) compared to the VEH group. The # represents a significant difference (#P < 0.05) compared to the naive group.

After retrieval, one-way ANOVA showed significant effects of treatment for pre-proBDNF (F_2,31_ = 3.71; *P* = 0.036; η^2^ = 0.19). As shown in Fig. 5A, the VEH and ROF 0.1 groups presented a significant reduction in pre-proBDNF than naive, suggesting that retrieval downregulates the pre-proBDNF expression within the DH. No significant differences in proBDNF (F_2,31_ = 0.95; *P* = 0.399; η^2^ = 0.06) and mBDNF (F_2,34_ = 0.57; *P* = 0.569; η^2^ = 0.03) were detected. As shown in Fig. 5B, C all groups presented similar expressions of proBDNF and mBDNF.

After Test A_1_, one-way ANOVA showed significant effects of treatment for proBDNF (F_2,30_ = 3.32; *P* = 0.050; η^2^ = 0.18). As shown in Fig. 5E, the ROF-treated group showed higher proBDNF expression than the VEH-treated group (*P* = 0.045), which might be involved in the ROF effects. No significant effects were detected for pre-proBDNF (F_2,30_ = 0.58; *P* = 0.565; η^2^ = 0.04) or mBDNF (F_2,30_ = 1.02; *P* = 0.374; η^2^ = 0.06). As shown in Fig. 5D and F, all groups presented similar levels of pre-proBDNF and mBDNF.

### The inhibition of PDE4 did not alter PKMζ expression in the DH after retrieval and Test A_1_

One-way ANOVA showed no significant effects of treatment for PKMζ expression after retrieval (F_2,34_ = 0.198; *P* = 0.821; η^2^ = 0.01) nor after Test A_1_ (F_2,31_ = 0.502; *P* = 0.610; η^2^ = 0.03). No significant differences were observed among groups for PKMζ expression (Figure S3), suggesting that PKMζ expression in the DH is not engaged in the behavioral effects induced by PDE4 inhibition.

## Discussion

Our results showed that (1) inhibiting PDE4 in the DH after a short retrieval reduced freezing behavior in Test A_2_, without changing fear expression during Test A_1_; (2) this effect depended on retrieval and Test A_1_ re-exposure and is spared by presenting a reminder foot shock; (3) The PDE4 inhibition after a short retrieval facilitates extinction in the next day; (4) PKA inhibition within the DH after retrieval or protein synthesis in the DH after Test A_1_ impaired the ROF-induced effect; (5) the systemic inhibition of PDE4 after retrieval produced similar effects and increased the expression of proBDNF in the DH without changing the pCREB/CREB ratio and PKMζ. These results contribute to the hypothesis that the PDE4 in the DH after a short retrieval may control the switch from reconsolidation to extinction in a new reexposure to the context.

Inhibiting PDE4 within the DH after retrieval did not change freezing behavior 24 h later (Test A_1_), suggesting a lack of treatment effect on reconsolidation, considering that reconsolidation-impairing or enhancing effects are short-term, i.e., observed 1 or 2 days after treatment during re-exposure to CS^20–27^. When animals were retested after 10 days (Test A_2_), the ROF-treated group presented a significant reduction in freezing behavior. Accordingly, a similar result was observed when ROF was given i.p. or into the infralimbic (IL) cortex 3 h after an extinction session^3^, suggesting that independent of retrieval length, inhibiting PDE4 impairs fear memory expression in a long-term manner. Importantly, when memory retrieval or Test A_1_ were omitted, the ROF-induced effects in Test A_2_ were abolished, suggesting that the inhibition of PDE4 activity after retrieval plus Test A_1_ exposure underlie the reduction of fear expression 10 days later. This effect could be attributed to a reduction in reactivated-memory persistence or extinction facilitation, since not only prolonged but repeated re-exposure to CS without US presentation can induce fear extinction and consequently reduce fear expression^19,28–30^. Our result favors the last interpretation because after receiving a reminder foot shock in Context C, the ROF-treated group reinstated fear expression. Moreover, when the extinction session was performed one day after retrieval, the ROF-treated group presented faster extinction than control either within and between sessions.

The role of PDE4 underpinning fear extinction has been reported^3,6,31–35^. When given 3 h after prolonged CS exposure, either systemically or in the IL cortex, ROF improved the persistence of extinction^3^, and rolipram (PDE4 inhibitor) facilitated extinction in the MPTP model of Parkinson's disease in mice^34^. However, the administration of rolipram impaired the extinction of a cued fear conditioning and fear-potentiated startle^33,35^. Moreover, reduced PDE4 activity in the dentate gyrus is involved in fear renewal^32^. Considering the selectivity of ROF and rolipram for PDE4 differs, it might contribute to the differences observed^36,37^.

Studies investigating the role of PDE4 subtypes in fear memory have shown distinct effects. For instance, PDE4B knockout mice presented no changes in cued fear memory formation^38^. In contrast, PDE4D knockout mice exhibited impaired fear memory retention^39^. However, PDE4B mutant mice, which have decreased activity of PDE4B, or PDE4D knockout that received an infusion of miRNA into the DH, displayed impairments retention of cued fear conditioning and improvement of inhibitory avoidance, respectively^40,41^. It is worth mentioning that ROF is a pan-PDE4 inhibitor, therefore the specific impact of distinct PDE4 subtypes in our condition requires further investigation.

Extinction facilitation was induced by inhibiting PDE4 after a short retrieval session associated with a CS exposure one day after CFC, a protocol commonly used to assess reconsolidation^19–22,24^. Of note, no effect was observed when ROF was given 5 min after a prolonged CS exposure^3^. Importantly, a classical mechanism induced by PDE4 inhibition is the enhancement of PKA signaling^42^. Indeed, PKA inhibition immediately after retrieval abolished the ROF-induced effect on Test A_2_. Of note, no per se effect of H89 was observed, which agrees with our previous study^3^. The PKA inhibition in the basolateral amygdala or in the DH of rats impaired fear memory reconsolidation^26,43^. Also, PKA inhibition reduced membrane hyperexcitability induced by fear memory retrieval in hippocampal slices^44^. However, in Lymnaea, PKA activity enhances when retrieval happens 6 h after conditioning, but not 24 h later^45^. In the extinction case, it is well-documented that PKA facilitates fear extinction of single prolonged stress, inhibitory avoidance, and CFC^11,12^. Here, the results suggest that PKA subserves the effects of PDE4 inhibition after contextual fear memory retrieval.

Protein synthesis triggered by Test A_1_ exposure is also involved in ROF effects. Indeed, protein synthesis underlies the fear memory extinction^46^. Importantly, no per se effect of ANI was observed. Accordingly, a study showed that the same dose of ANI in the DH was not able to induce any per se effect but reversed the memory-improvement effects induced by spermidine^23^. Then, it is conceivable to suggest that PDE4 activity within the DH after a short retrieval session controls the memory’s fate in the next CS exposure; when its activity is inhibited, it favors extinction instead of reconsolidation.

Memory retrieval enhanced the pCREB/CREB ratio in the DH 90 min after treatment. Accordingly, an enhancement in pCREB/CREB, in the DH was shown after a short retrieval session of animals exposed to cued or CFC, and inhibitory avoidance^47–50^. In contrast, a further enhancement of pCREB/CREB was not detected in the ROF-treated group^13,51^. The enhancement of pCREB/CREB after prolonged exposure to CS and extinction has been debated. For instance, extinction was not sufficient to enhance the pCREB/CREB ratio in the DH^47^. Thus, considering that ROF treatment facilitated extinction instead of reconsolidation, the lack of effect on pCREB/CREB ratio would be expected. However, enhancing pCREB activity in the DH facilitated fear extinction in rats submitted to CFC^52^. Differences in protocols, including the moment of pCREB analysis, which varies between 30 min and 9 h after retrieval, or the use of western blot analysis instead of immunohistochemistry, may account for these discrepancies^53^. After Test A_1_, no significant differences were observed in the pCREB/CREB ratio. However, the VEH group showed higher pCREB expression compared to the naive group. Considering that Test A_1_ is also a retrieval session, this result agrees with findings showing an enhancement in pCREB expression in the DH during reconsolidation^47–50^, which is not observed in the ROF-treated group. Then, our results agree with a study showing an increase of pCREB-positive cells in DH during reconsolidation, but not in the intermediate phase between reconsolidation and extinction (limbo) or extinction groups^50^. Thus, suggesting that PDE4 activity after a short retrieval might be involved in the dynamic between extinction and reconsolidation.

After retrieval, no changes in the pre-pro, pro, or mBDNF were detected in VEH or ROF-treated groups. Accordingly, no role for BDNF in the DH after a short retrieval session has been suggested^9^. In addition, ROF administration after a prolonged retrieval enhanced the pre-proBDNF expression in the DH and IL cortex^3,31^. Here, the treatment with ROF after retrieval enhanced proBDNF expression after Test A_1_. ProBDNF binding to the P75NTR receptor induces long-term depression (LTD) in hippocampal neurons^54^, a mechanism that underlies the induction of fear extinction^55,56^. Evidence suggests that proBDNF in the DH facilitates extinction^16,57^ and that proBDNF/p75NTR signaling in the amygdala and hippocampus plays a pivotal role in LTD and fear extinction^55,58^. Thus, our findings further suggest that in the ROF-treated group, Test A_1_ exposure is sufficient to induce fear extinction. BDNF activity through TrkB activation sustains late-LTP through PKMζ activity even when protein synthesis is inhibited^17^. Here, no changes in PKMζ expression in the DH after retrieval or after Test A_1_ were observed, indicating that PDE4 inhibition effects may not be related to memory persistence mechanisms.

Altogether, our results suggest that PDE4 activity in the DH after retrieval controls the transition from reconsolidation to extinction. These findings may shed light on the development of new treatments and behavioral strategies to attenuate maladaptive memories related to psychiatric disorders such as PTSD.

## Methods

### Animals

Male Wistar rats (3 months old, 300–350 g) were provided by the Biological Sciences Sector of the Federal University of Parana (UFPR). Animals were housed in groups of four in polycarbonate cages (48 × 37 × 21 cm) with food and water at libitum, under controlled temperature (22 ± 2 °C) in a 12 h light–dark cycle (lights on 07:00 AM). All procedures were approved by the Ethical Committee for the Use of Animal in Experimentation (CEUA/UFPR authorization number #1318), following Brazilian legislation^59^. All procedures and methods were performed according the ARRIVE essential guidelines.

### Stereotaxic surgery

Animals were deeply anesthetized with ketamine (100 mg/ml; Syntec®, Brazil) and xylazine (10 mg/ml; Syntec®, Brazil) and were submitted to stereotaxic surgery to implant bilateral guide-cannulas aiming at the DH, following the same procedures previously described^20,21,60^. Figure S1 represents the DH slices marked with methylene blue infused to check the infusion site. Only animals with bilateral injection into the DH (AP: from − 4.2 to − 3.3; ML: from 1.5 to 3.0; DV: from − 2.5 to − 3.0) were included in the statistical analysis.

### General behavioral procedures

Ten days after surgery recovery, the animals underwent contextual fear conditioning (CFC)^19,20^. All experiments were conducted between 1 and 6 pm. CFC was performed in Context A, a rectangular chamber (35 × 20 × 30 cm) with aluminum sidewalls, a front wall and ceiling door made of transparent Plexiglass, and a grid floor made of stainless-steel bars, connected to a circuit board and a shock generator (Insight®, Brazil). Animals individually underwent familiarization in Context A (3 min). On the next day, each rat was exposed to Context A for fear conditioning, consisting of initial 30 s, 3-foot shocks (unconditioned stimulus; US) of 0.8 mA/3 s with intervals of 30 s, and final 30 s. After 24 h, animals were exposed to a short retrieval session (3 min) in Context A without US presentation. The treatments were administered 5 min after this session. After 1 and 10 days, animals were exposed to Context A in Test A_1_ and Test A_2_ (3 min), respectively, to evaluate the treatment effects. Fear memory generalization was evaluated by exposing the animals to a neutral Context B (3 min; transparent acrylic box covered with black lid; 34 × 26 × 33 cm) one day after Test A_1_ and Test A_2_.

Moreover, Context B was also used to avoid memory reactivation. In this case, animals were exposed to Context B for 3 min, and sessions were called no-retrieval (when retrieval was omitted) or no-recall (when Test A1 was omitted) sessions.

When appropriate, an extinction session was conducted by exposing the animals to Context A for 20 min. This session was divided into early extinction, i.e., the first 3 min, and late extinction, i.e., the last 3^22^ or in 2-min blocks. When necessary, memory reinstatement was performed 24 h after extinction. The animals were exposed to Context C (Context A modified with different colors, textures, and cues) for a reminder foot shock (0.5 mA), and a reinstatement test was conducted after 24 h, consisting of a 3 min reexposure to Context A^22^_.._

Freezing behavior, characterized by the lack of movements excluding those involved with breathing^61^, was measured as a fear memory retention parameter^3,19,20,60^. The measures were evaluated manually using a stopwatch by a trained experimenter blind to the treatments.

### Drugs and intra-DH infusion

Roflumilast (ROF; PDE4 inhibitor; 9 ng/0.5 µL/side or 0.1 mg/kg i.p.; a gift from Maastricht University, The Netherlands)^3^ was solubilized in 0.9% NaCl solution containing 10% polyoxyethylene Sorbitan monooleate (Tween-80®, Sigma-Aldrich, USA). Anisomycin (ANI; protein synthesis inhibitor; 2 µg/0.5 µL/side; Sigma-Aldrich, USA)^23^ was dissolved in 1 M HCl diluted with PBS. The pH was adjusted to 7.4 using NaOH 1 M. H89 (PKA inhibitor; 10 µM/0.5 µL/side; Sigma-Aldrich, USA)^3^ was dissolved in 0.9% NaCl solution containing 10% dimethylsulfoxide. The control groups received the vehicle solution (VEH) of each drug. The selection of doses was based on previous studies where ROF interfered with fear memory extinction^3^. H89 and ANI doses were based on studies showing that they did not exert per se effects but were able to prevent effects mediated by other treatments^3,23^.

Bilateral infusions into the DH were performed using a needle (0.3 mm in diameter and 10.5 mm in length) into a polyethylene tubing connected to a microsyringe. During 30 s, 0.5 µL/side of either VEH or drug was injected using two 5.0 µL syringes connected to an infusion pump (Insight, Brazil).

### Western blot

The DH samples of independent groups of animals were collected 90 min after retrieval or Test A_1_. A naive group was used to record the basal expression of proteins. The DH was dissected from slices obtained from bregma at AP − 3.0 to − 4.5 mm. Protein lysates were prepared in a solution containing a loading buffer with phosphatase and protease inhibitors. 30 µg of total protein was loaded and resolved in SDS-PAGE. 10% polyacrylamide gel was used for PKMζ, pCREB, and CREB and 14% for BDNF. The proteins were transferred onto nitrocellulose membranes and blocked for 1 h at room temperature (Odyssey blocking buffer; Li-Cor®, Lincoln, USA) diluted 1:1 in tris-buffered saline (TBS). Primary antibody incubation with rabbit anti-BDNF (1:1000; Abcam; ab108319), rabbit anti-PKMζ (1:1000; Abcam; ab59364), rabbit anti-pCREB, mouse anti-CREB (1:1000; Cell Signaling; #9198S and #9104) or mouse anti-GAPDH (1:2,000,000; Fitzgerald Industries; #10R-G109A) was done overnight at 4 °C. Membranes were washed and incubated with the secondary antibodies donkey anti-mouse IRDye 680 or goat anti-rabbit IRDye 800 (both 1:10,000, Li-Cor®) for 1 h at room temperature. After washing, the membranes were dried and scanned by Odyssey CLx Infrared Imaging System (Li-Cor®). This provides two-wavelength scanning since the secondary emits signals detected in different wavelengths (red or green), consequently allowing the analysis of different target proteins in the same membrane, following the animal species used as background for the primary antibody. Protein bands were quantified using ImageJ (NIH, USA). The target signal obtained was normalized to GAPDH to control loading differences.

### Statistics

The sample size was determined a priori by G-power® (Kiel University, Germany) as eight animals per group (α = 0.05; β = 0.20; η^2^ = 0.14). Group sizes were equal by design but were unequal in a few cases due to experimental loss (infusion site outside of the DH). After assuring data normality and homogeneity, behavioral analysis was subjected to student’s t-test, repeated-measures, two-way repeated-measures, or one-way ANOVA according to each experiment. Western blot was analyzed by one-way ANOVA. The statistical significance level was set as P ≤ 0.05. In all cases, the Newman-Keuls post-hoc was established for multiple comparisons. Grubb’s test (P ≤ 0.05) was applied for outlier exclusion. The data is represented as mean ± SEM and the individual values. Statistica 12 (StatSoft, USA) and GraphPad Prism 8 (GraphPad Prism, USA) were used for statistics and graphing, respectively. The partial eta squared (η^2^) indicates the effect size, considering η^2^ = 0.14 a large effect^62^.

### Supplementary Information

Supplementary Information.

## Data Availability

All data that support this study are available from the corresponding author upon request.
