# Supplementary material for: Phosphodiesterase 4 inhibition after retrieval switches the memory fate favoring extinction instead of reconsolidation
Source: Sci Rep. 2023 Nov 21;13:20384. doi: 10.1038/s41598-023-47717-1 (PMC10663466; doi:10.1038/s41598-023-47717-1)
Supplement: Supplementary file 1 — Supplementary Information. [file 41598_2023_47717_MOESM1_ESM.docx]

**Phosphodiesterase 4 inhibition after retrieval switches the memory’s fate**

**favoring extinction instead of reconsolidation**

Jeferson Machado Batista Sohn, Msc; Nathalie Carla Cardoso; Ana Maria Raymundi, PhD; Jos Prickaerts, PhD; Cristina Aparecida Jark Stern*, Ph.D.


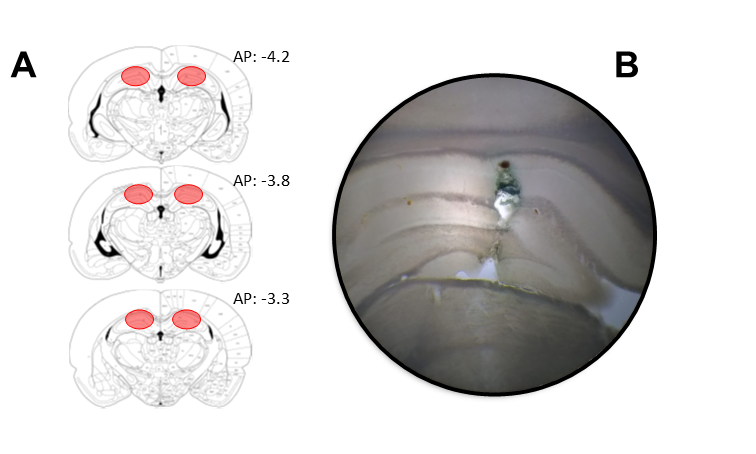
**Figure S1. Representative site of drug injection. A.** Schematic drawing of the CA1 region of the DH adapted from Paxinos and Watson, 2009. AP: from -4.2 to -3.3; ML: from 1.5 to 3.0; DV: from -2.5 to -3.0. **B.** Representative infusion site placement in the DH – 40x amplification. Animals were included in the analysis when the treatment was bilaterally infused into the DH.

**Table S1. PDE4 inhibition did not induce changes in memory generalization.**

| **PDE4 inhibition in the DH after retrieval** | | | | |
| --- | --- | --- | --- | --- |
| Repeated-measures ANOVA  F_1, 16_=0.09; *P*=0.76 | VEH | | ROF 9 ng | |
|  | *n* | Mean ± SEM | *n* | Mean ± SEM |
| % freezing test B1 | 9 | 5.31 ± 1.62 | 9 | 1.79 ± 0.48 |
| % freezing test B2 | 9 | 7.65 ± 2.24 | 9 | 3.33 ± 1.15 |
| **PDE4 inhibition in the DH without retrieval** | | | | |
| Repeated-measures ANOVA  F_1, 16_=0.06, *P*=0.80 | VEH | | ROF 9 ng | |
|  | *n* | Mean ± SEM | *n* | Mean ± SEM |
| % freezing test B1 | 9 | 7.41 ± 1.84 | 9 | 10.06 ± 5.54 |
| % freezing test B2 | 9 | 2.16 ± 0.97 | 9 | 3.15 ± 1.94 |
| **PDE4 inhibition after retrieval without test A1 recall** | | | | |
| Repeated-measures ANOVA  F_1, 14_=0.17, *P*=0.69 | VEH | | ROF 9 ng | |
|  | *n* | Mean ± SEM | *n* | Mean ± SEM |
| % freezing test B1 | 8 | 10.14 ± 3.02 | 8 | 9.86 ± 3.13 |
| % freezing test B2 | 8 | 7.92 ± 3.12 | 8 | 9.38 ± 2.61 |

**Figure S2. Systemic PDE4 inhibition resulted in effects comparable to those observed with intra-DH administration.** The experimental design adopted has been represented above the graphs. The purple arrow represents the treatment i.p. with ROF or VEH, 5 min after the sessions. **A**) PDE4 inhibition did not induce any effects during the retrieval and test A1 sessions. However, animals that received ROF 0.1 mg/kg presented less freezing behavior than controls in test A2. **B**) The omission of context A, 24 h after the retrieval and treatments, abolished the effects of PDE4 inhibition effects in test A2. The data is represented by mean 士 S.E.M. and the individual values of the percentage of freezing expressed by animals during each session. The * represents a significant difference compared to VEH in the same session. n: ROF=9; VEH=9.


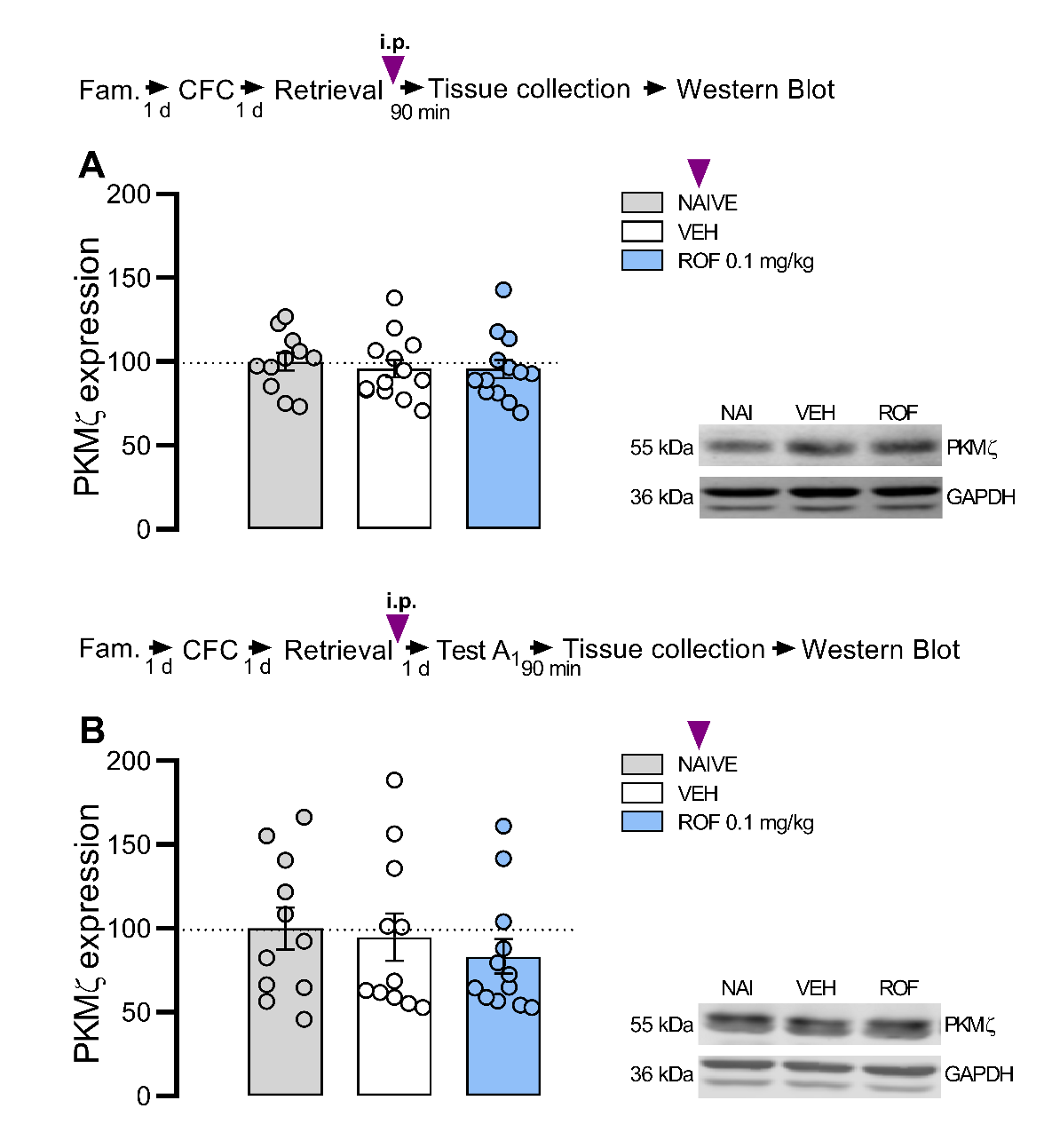


**Figure S3. Effects of systemic PDE4 inhibition on total PKMζ expression in the DH.** The experimental design adopted has been represented above the graphs. The purple arrow represents the treatment i.p. with ROF 0.1 mg/kg or VEH, 5 min after retrieval session. On the right side of the graphs are the representative bands on the western blots for PKMζ and GAPDH expression. **A**) PDE4 inhibition did not change the PKMζ expression 90 min after retrieval session in the DH. n: NAI=11 ROF=13; VEH=13. **B**) PDE4 inhibition did not change the PKMζ expression 90 min after test A1 in the DH. n: NAI=11 ROF=11; VEH=12. The data is represented by mean 士 S.E.M. and the individual values of the percentage PKMζ expression in relation to the Naive group.

**
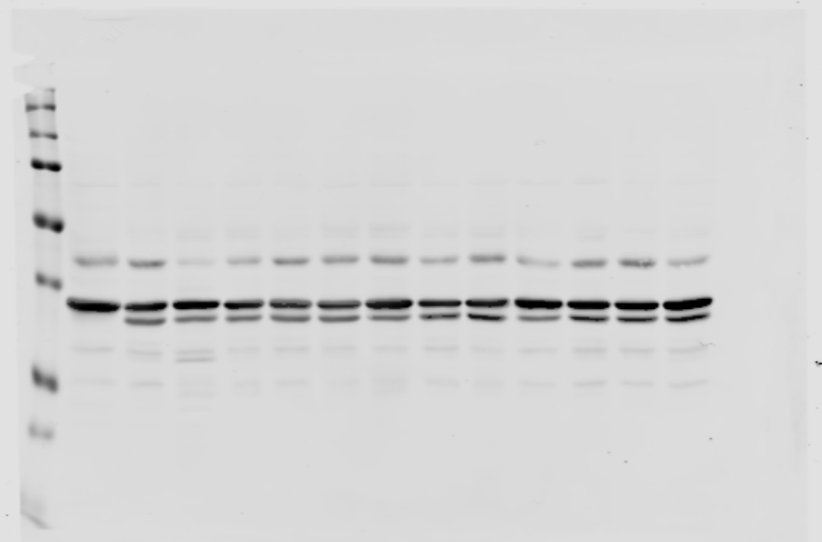

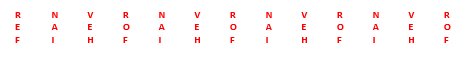

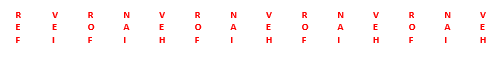

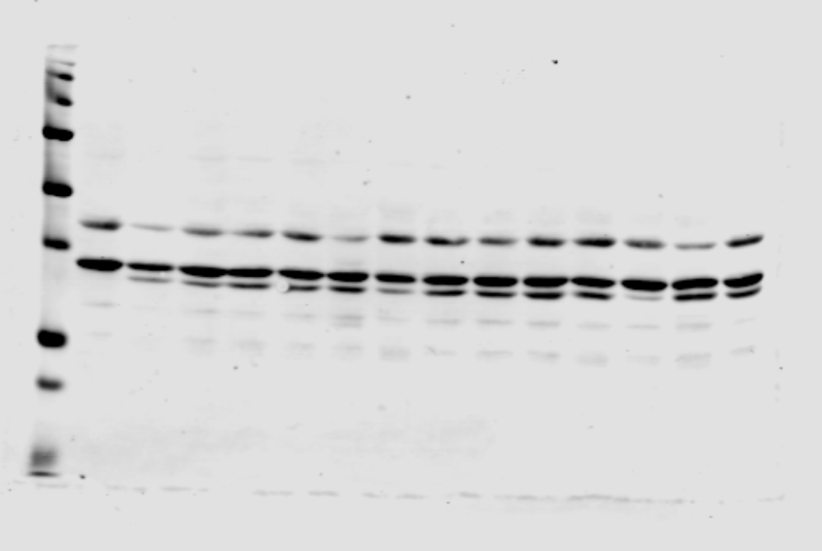

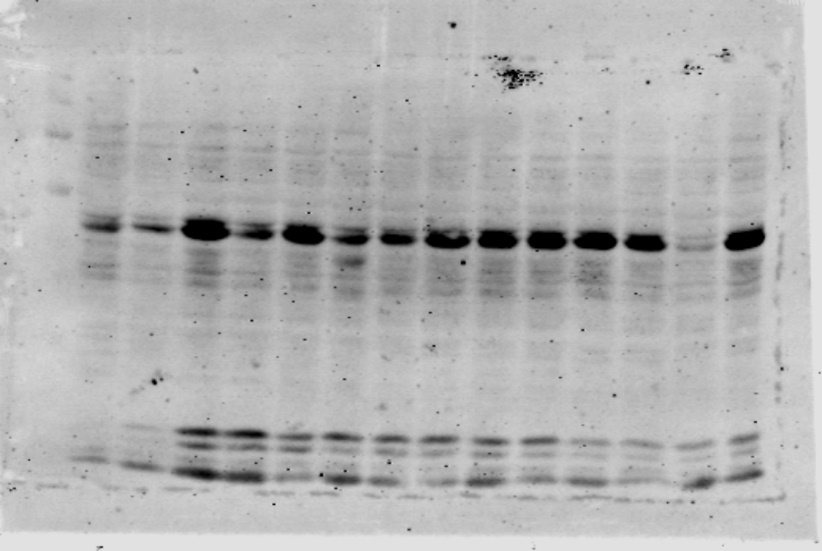

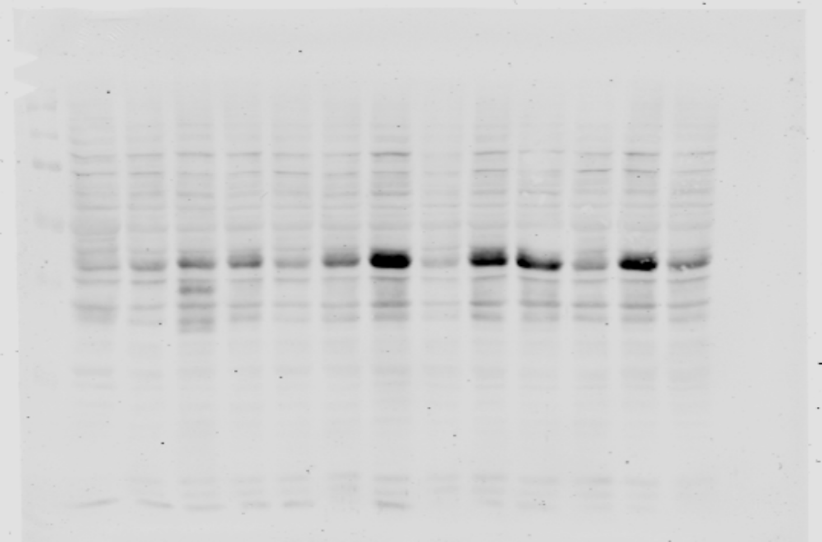

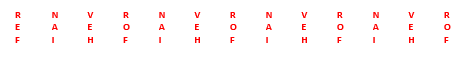
**

**36 kDa**

**43 kDa**

**36 kDa**

**43 kDa**

**36 kDa**

**43 kDa**

**43 kDa**

**43 kDa**

**43 kDa**

Green Signal

Red Signal

**43 kDa**

**36 kDa**

**43 kDa**

**36 kDa**

**43 kDa**

**36 kDa**

**
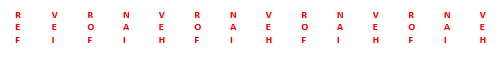
**

**
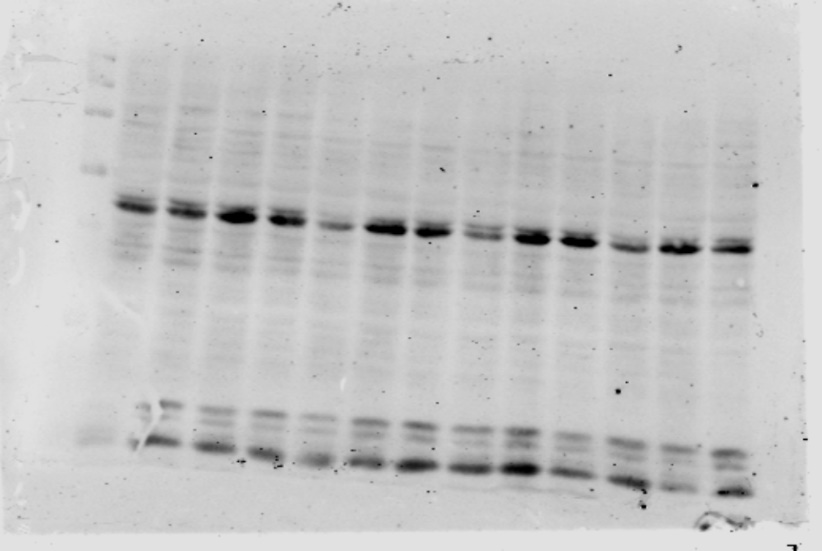
**

**
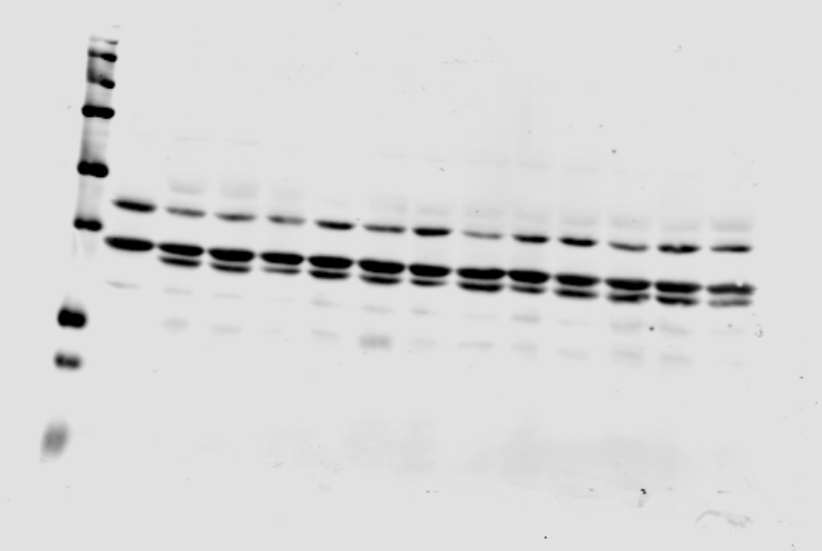
**

**
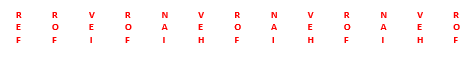
**

**
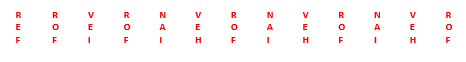
**

**Figure S4. Western blot membranes of pCREB and pCREB expression in the DH after fear memory retrieval.** On the left side is the green signal (800 nm) scanning showing pCREB expression (43 kDa). On the right side is the red signal (680 nm) scan showing CREB and GAPDH expression (43 and 36 kDa). The sample load in each slot is represented. REF is a hippocampus sample run in all the gels in order to control the scan intensity of the membranes. The red boxes represent the representative cropped bands related to the results expressed in Figure 4A-C.


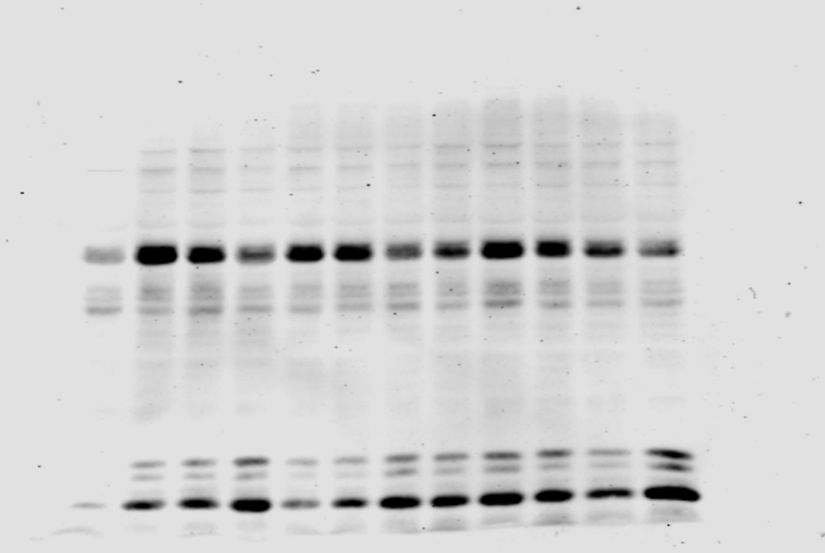

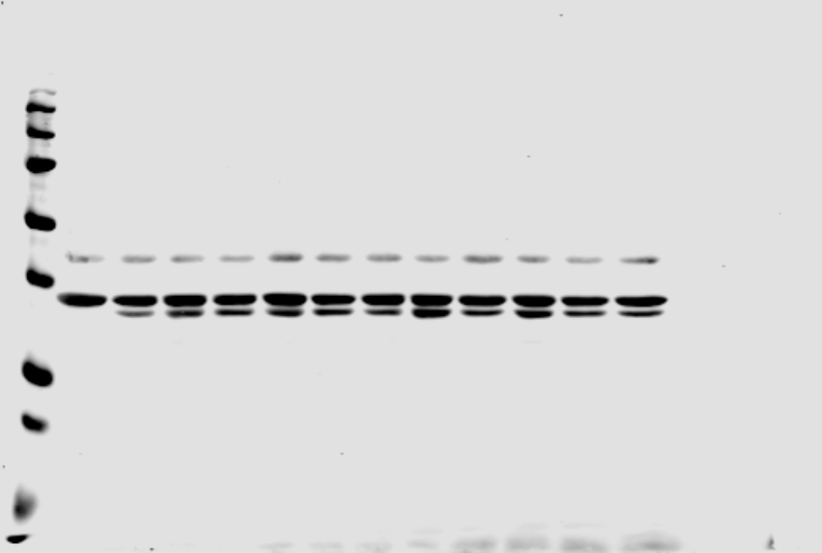


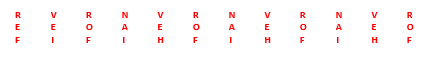


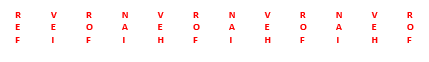


**43 kDa**

**43 kDa**

**36 kDa**


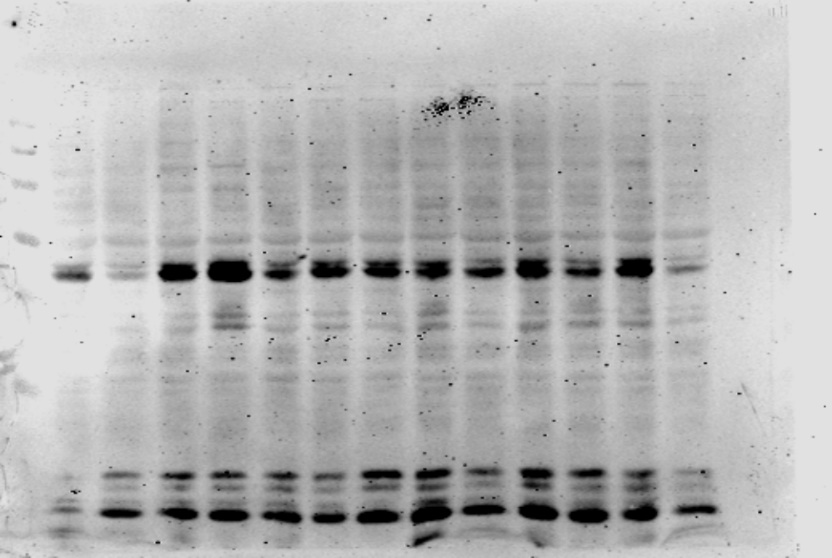

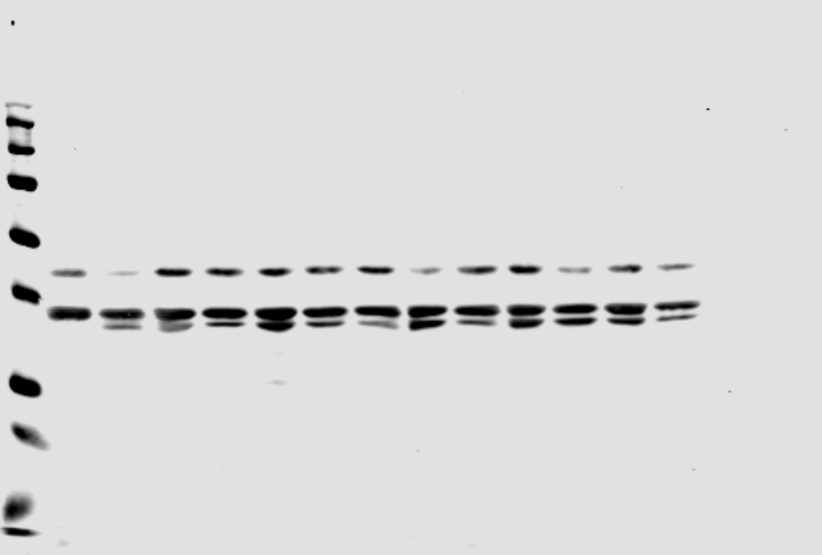


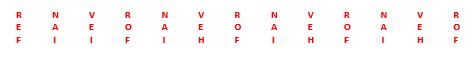

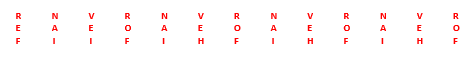


**43 kDa**

**43 kDa**

**36 kDa**


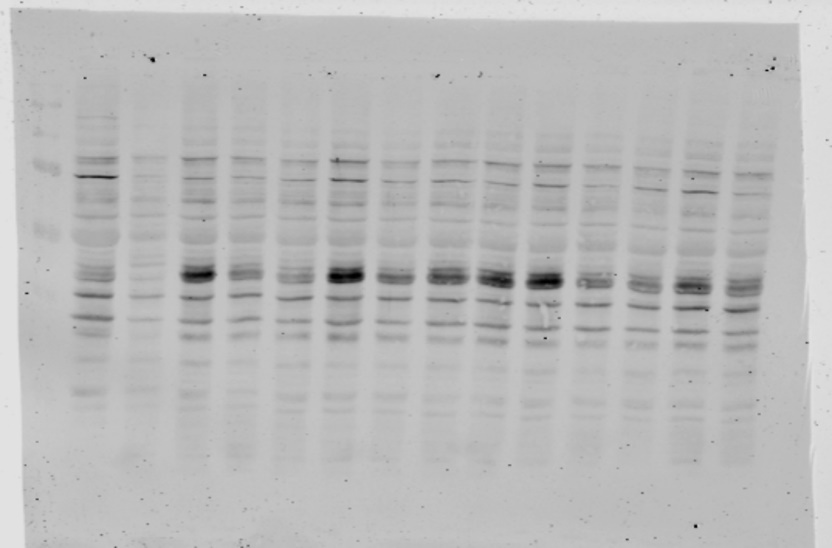


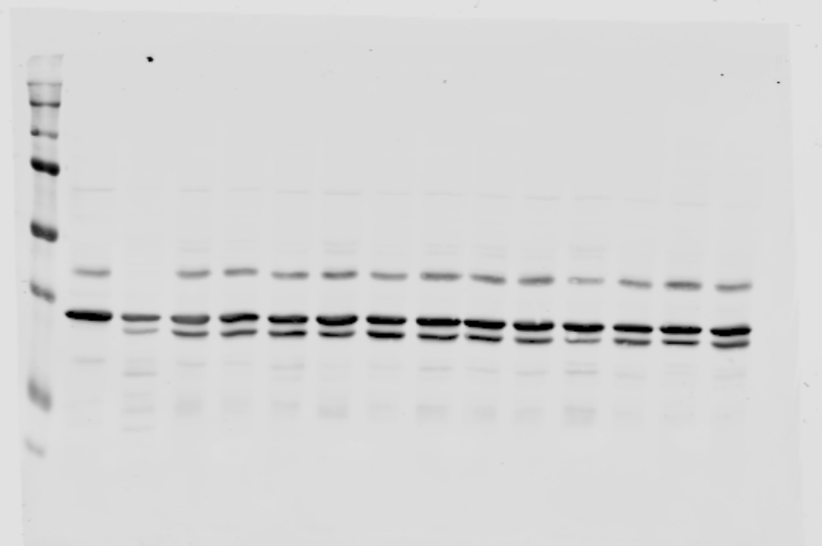


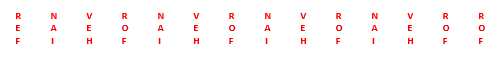


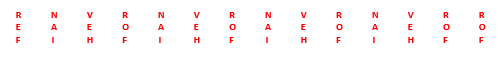


**43 kDa**

**36 kDa**

**43 kDa**

**Figure S5. Western blot membranes of pCREB and pCREB expression in the DH after Test A_1_.** On the left side is the green signal (800 nm) scanning showing pCREB expression (43 kDa). On the right side is the red signal (680 nm) scan showing CREB and GAPDH expression (43 and 36 kDa). The sample load in each slot is represented. REF is a hippocampus sample run in all the gels in order to control the scan intensity of the membranes. The red boxes represent the representative cropped bands related to the results expressed in Figure 4D-F.


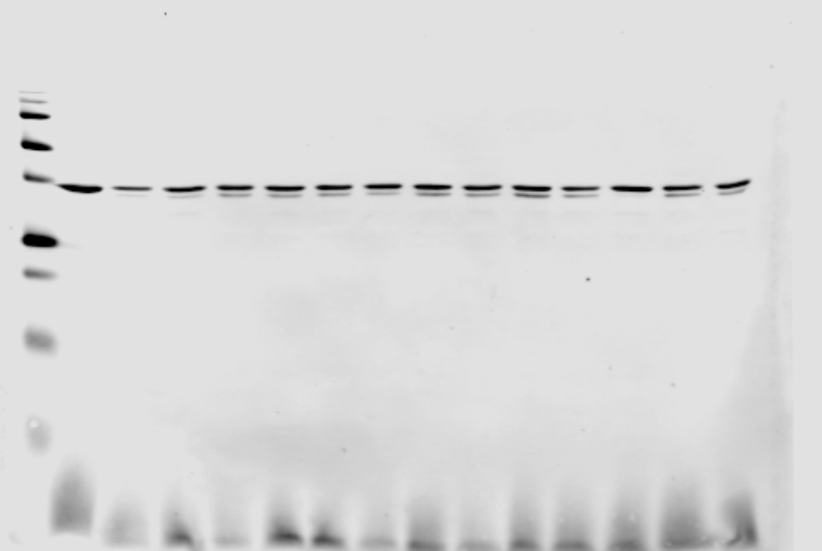

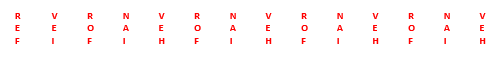

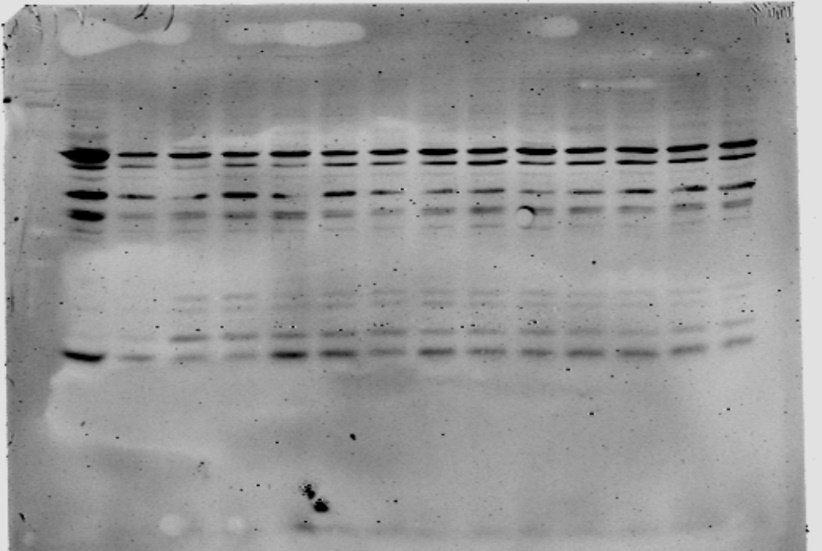


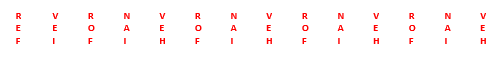


**32 kDa**

**36 kDa**

**16 kDa**

**14 kDa**


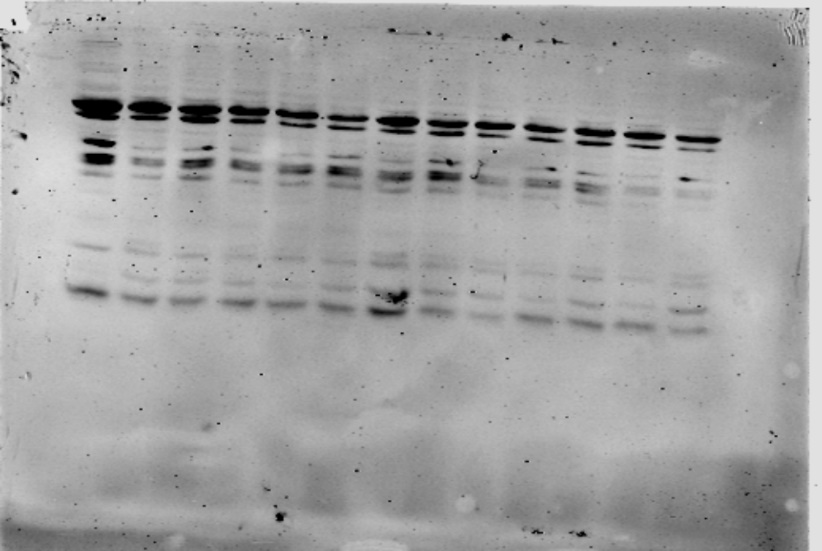


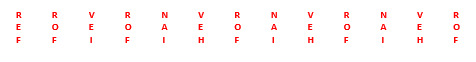

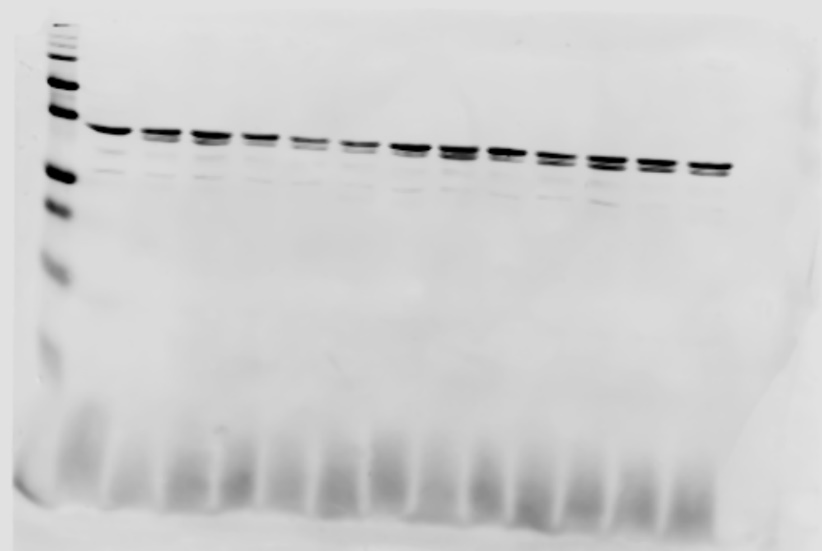

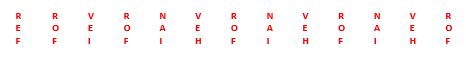


**32 kDa**

**36 kDa**

**16 kDa**

**14 kDa**


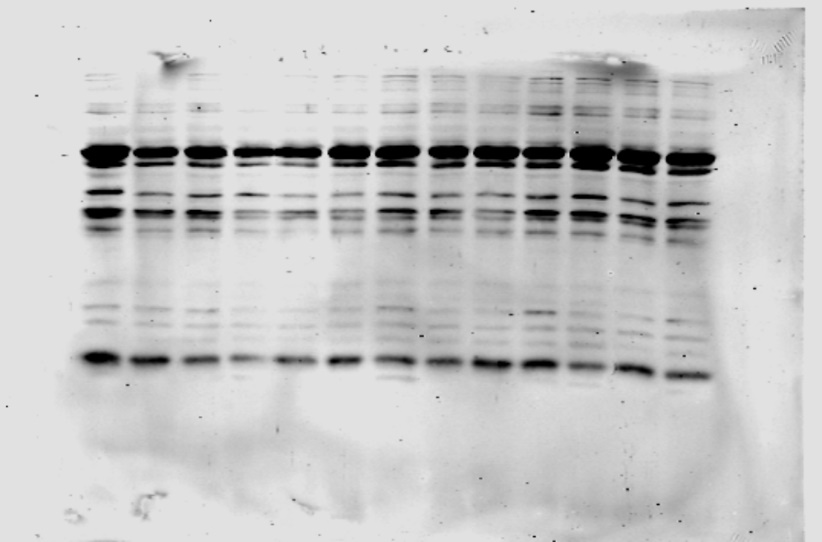


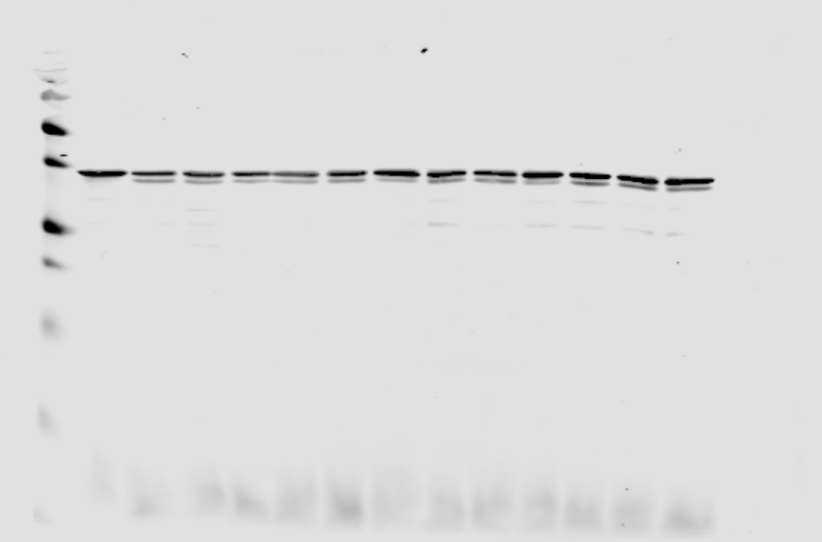

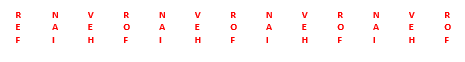


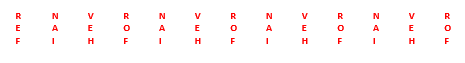


**36 kDa**

**32 kDa**

**16 kDa**

**14 kDa**

**Figure S6. Western blot membranes of BDNF expression in the DH after fear memory retrieval.** On the left side is the green signal (800 nm) scanning showing BDNF expression (32, 16 and 14 kDa). On the right side is the red signal (680 nm) scan showing GAPDH expression (36 kDa). The sample load in each slot is represented. REF is a hippocampus sample run in all the gels in order to control the scan intensity of the membranes. The red boxes represent the representative cropped bands related to the results expressed in Figure 5A-C.


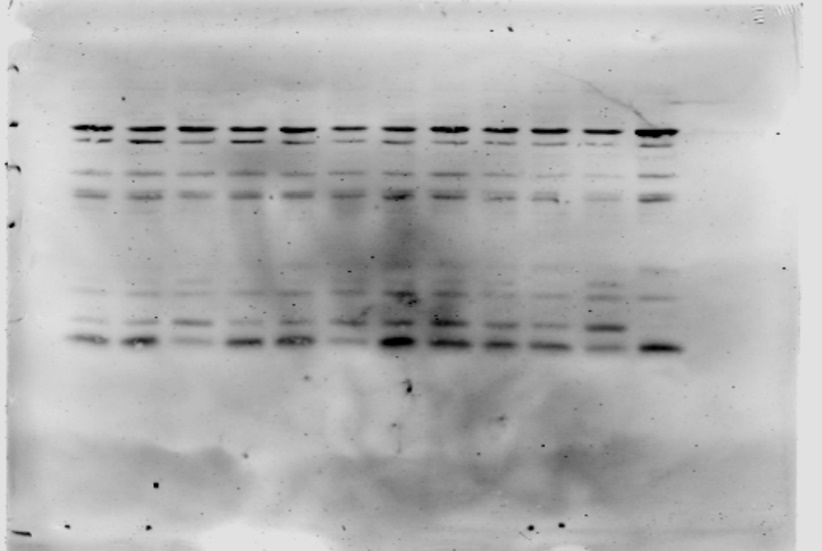

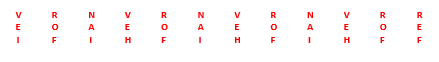

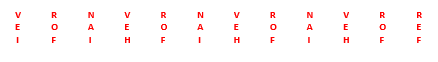

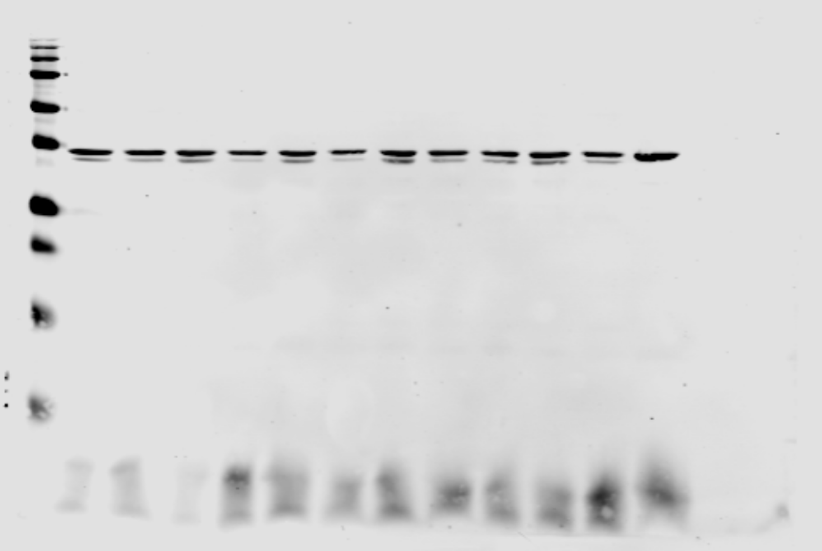


**36 kDa**

**32 kDa**

**14 kDa**

**16 kDa**


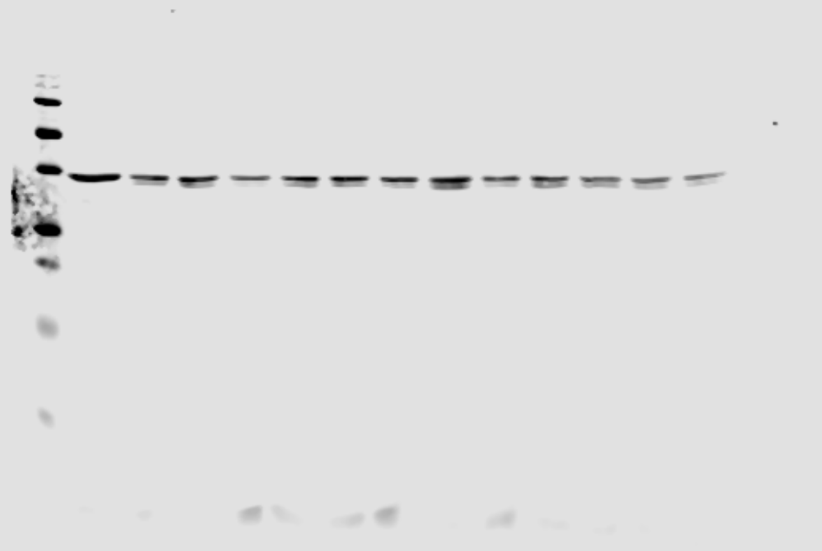

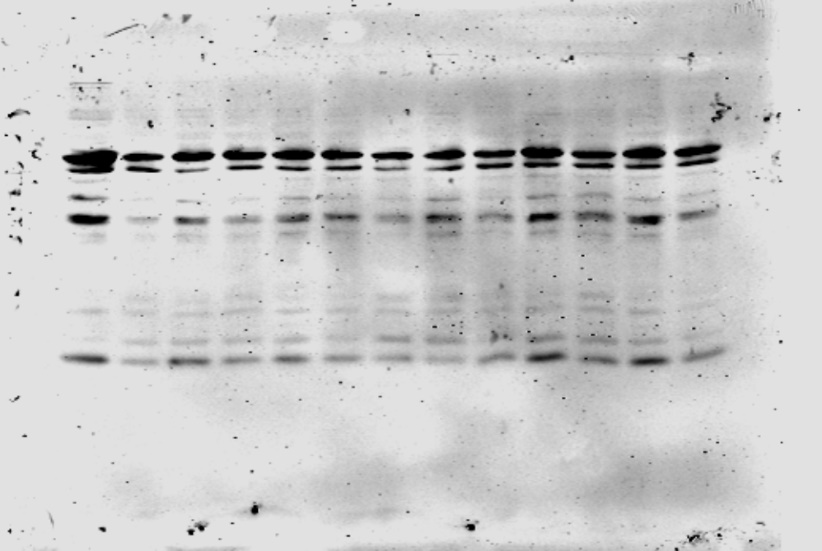


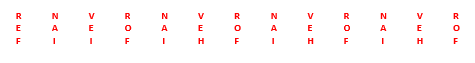


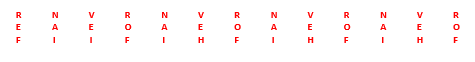


**36 kDa**

**32 kDa**

**14 kDa**

**16 kDa**


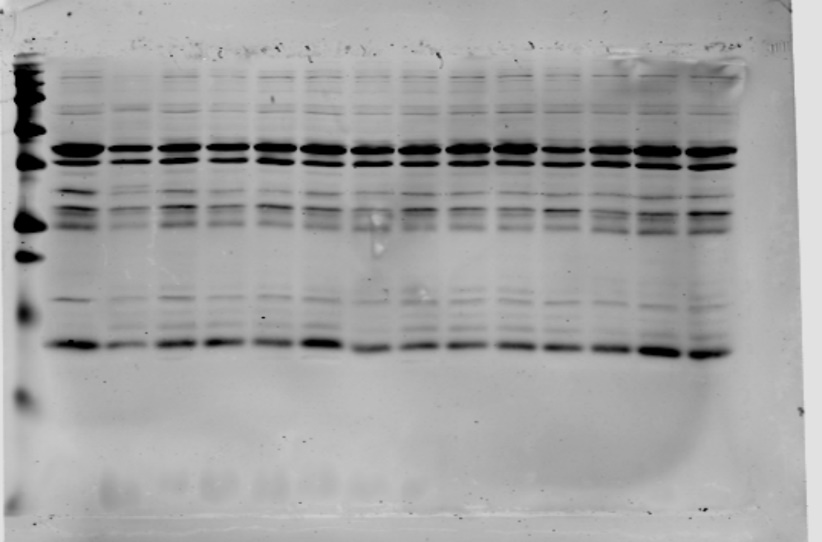

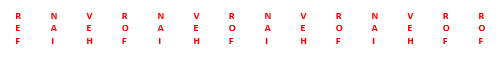

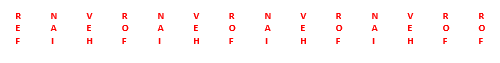

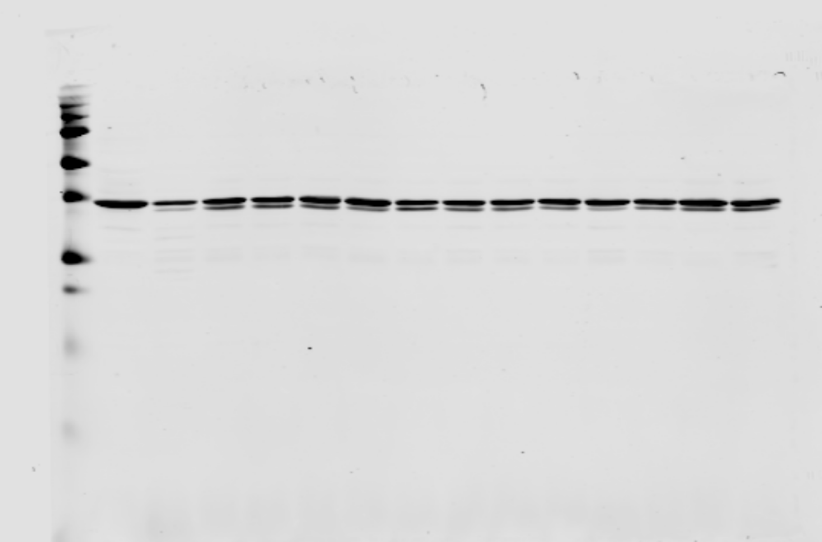


**36 kDa**

**32 kDa**

**16 kDa**

**14 kDa**

**Figure S7. Western blot membranes of BDNF expression in the DH after Test A_1_.** On the left side is the green signal (800 nm) scanning showing BDNF expression (32, 16 and 14 kDa). On the right side is the red signal (680 nm) scan showing GAPDH expression (36 kDa). The sample load in each slot is represented. REF is a hippocampus sample run in all the gels in order to control the scan intensity of the membranes. The red boxes represent the representative cropped bands related to the results expressed in Figure 5D-F.


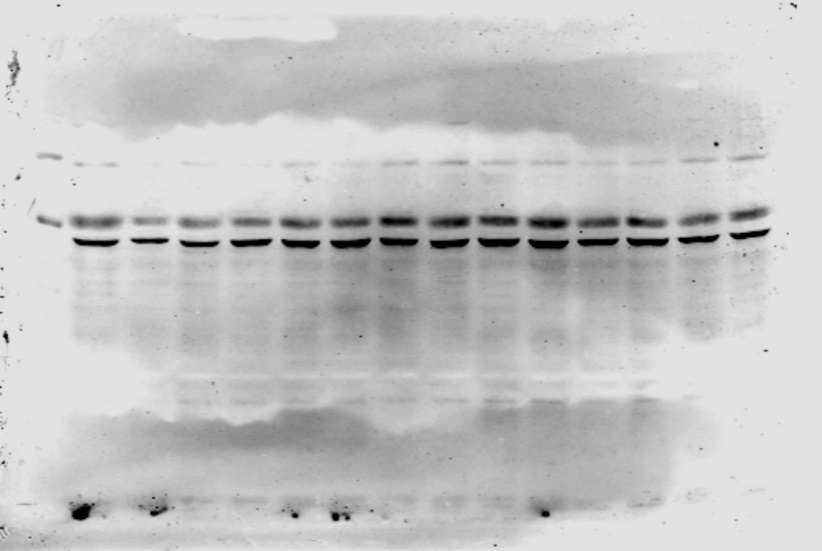

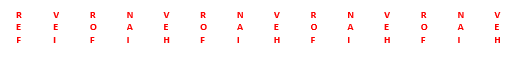

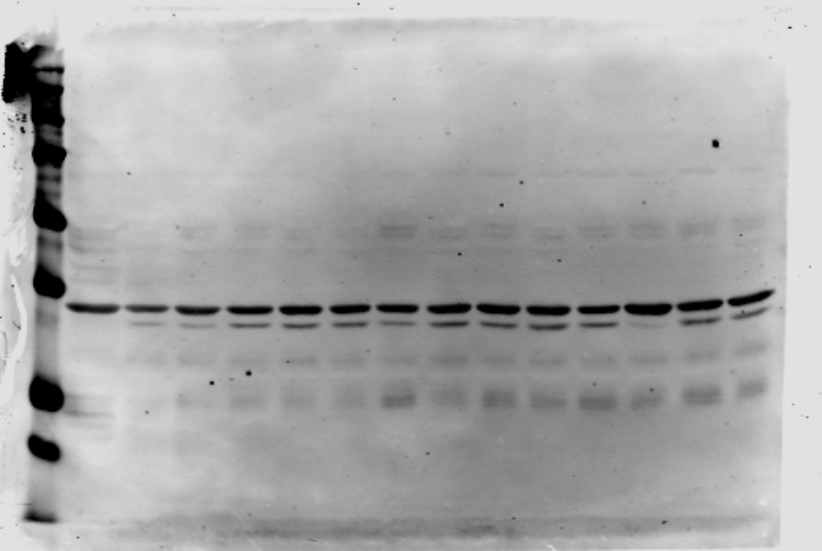


**55 kDa**


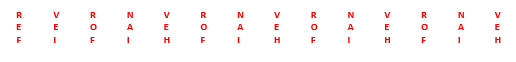


**36 kDa**


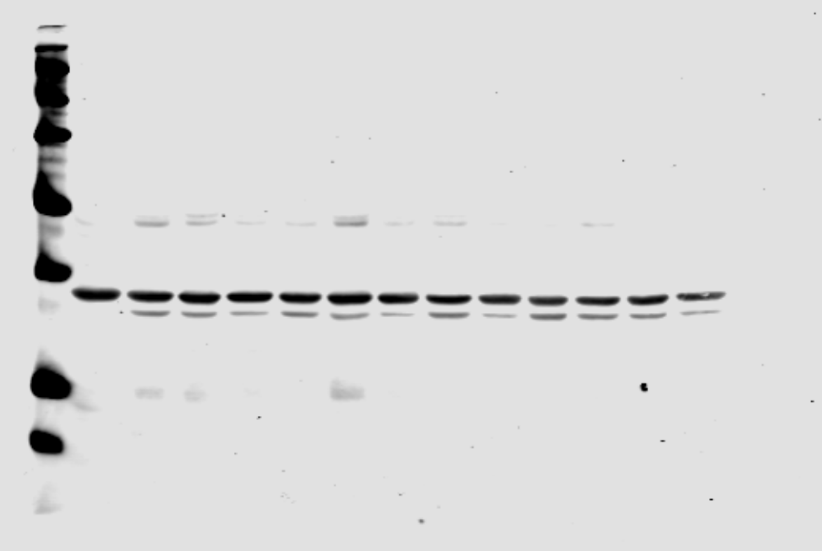

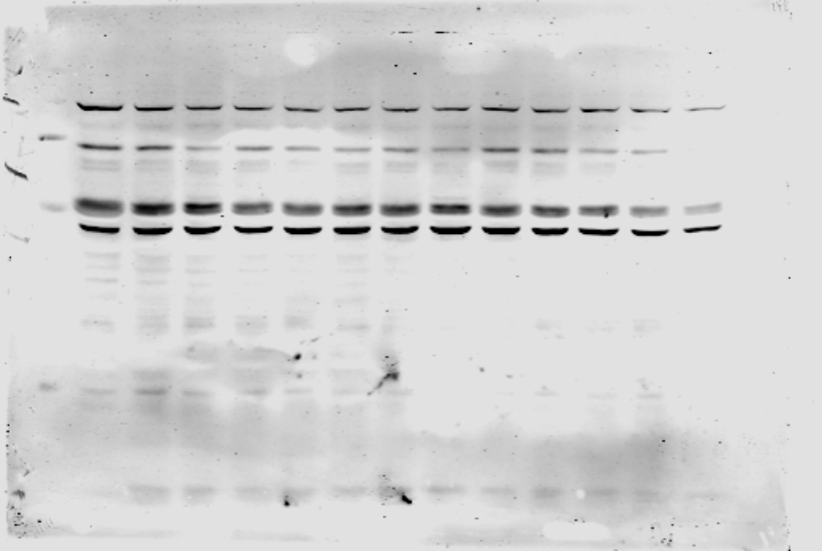


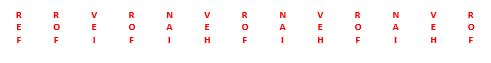


**55 kDa**


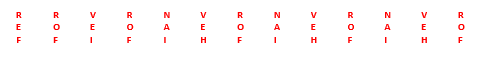


**36 kDa**


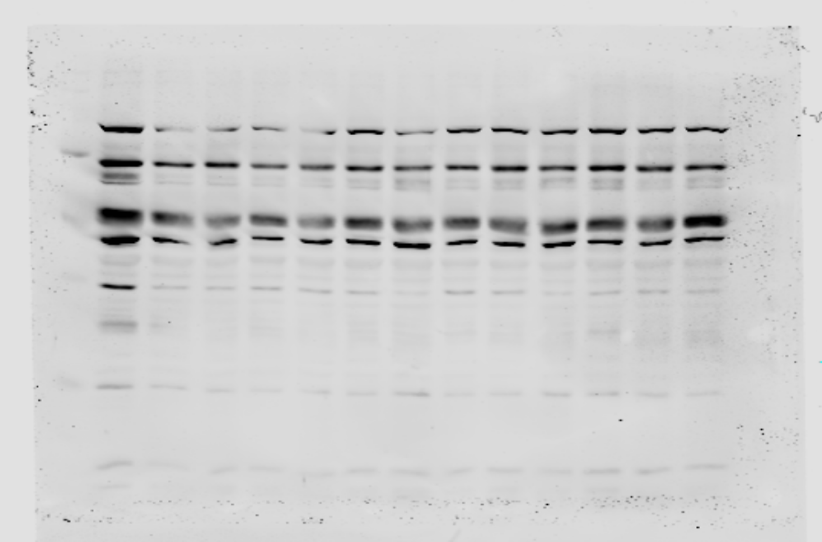


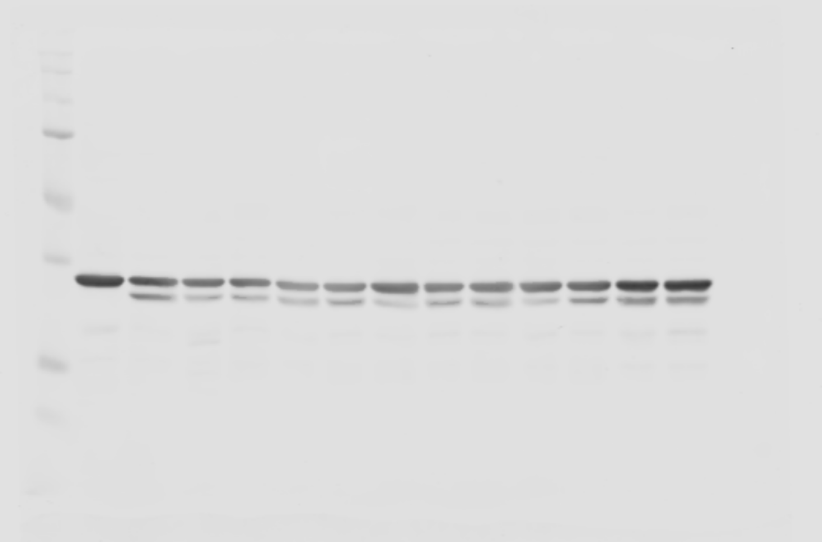

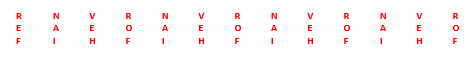


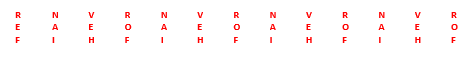


**55 kDa**

**36 kDa**

**Figure S8. Western blot membranes of PKMζ expression in the DH after fear memory retrieval.** On the left side is the green signal (800 nm) scanning showing PKMζ expression (55 kDa). On the right side is the red signal (680 nm) scan showing GAPDH expression (36 kDa). The sample load in each slot is represented. REF is a hippocampus sample run in all the gels in order to control the scan intensity of the membranes. The red boxes represent the representative cropped bands related to the results expressed in Figure S3A.


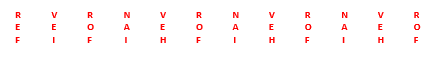

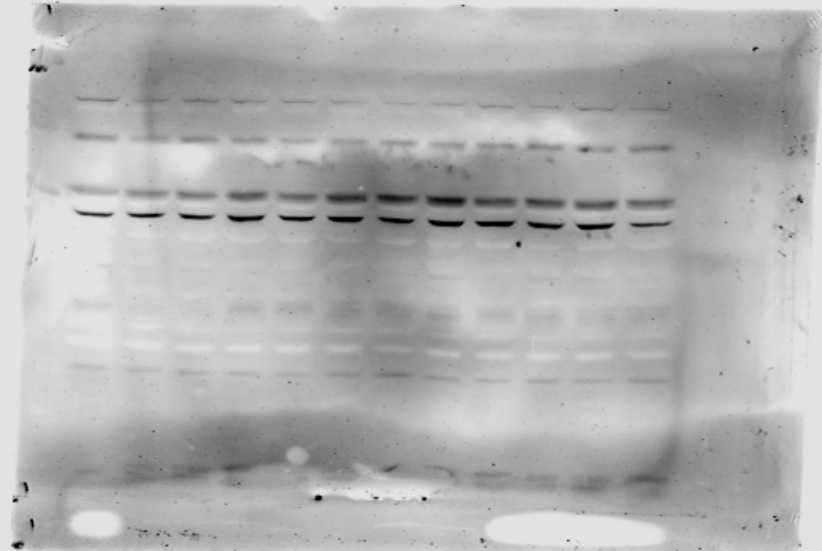

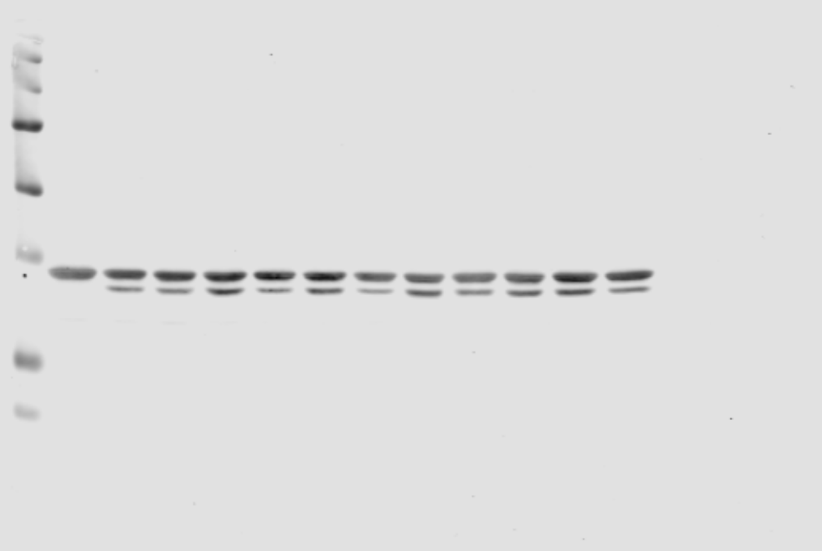


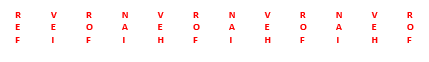


**55 kDa**

**36 kDa**


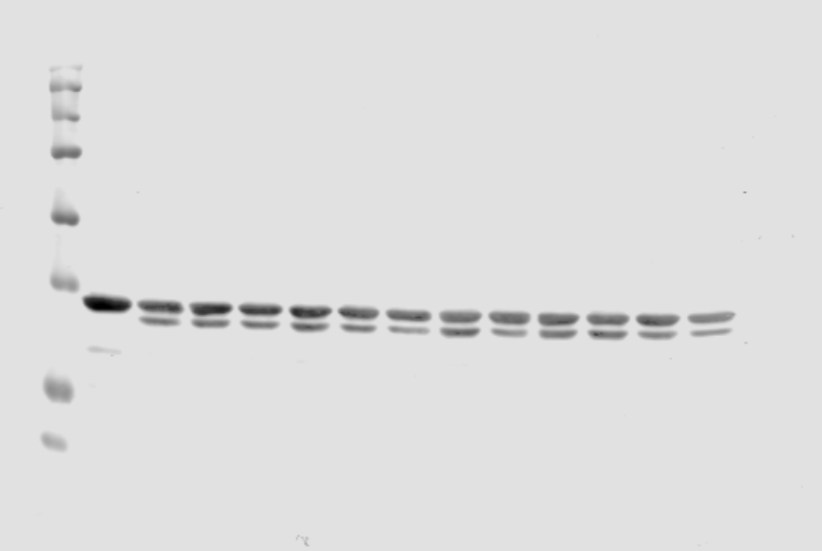

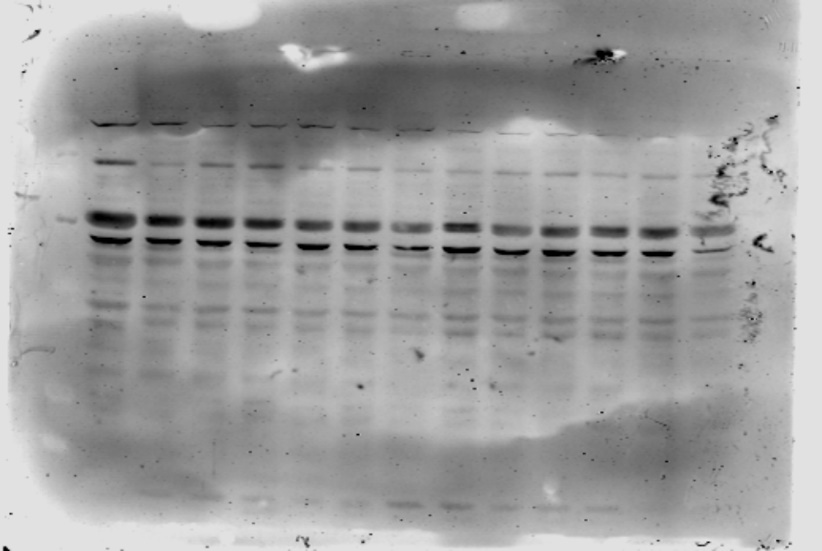


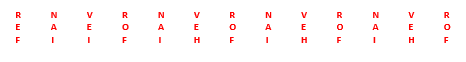


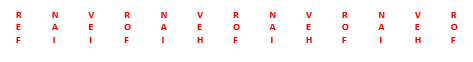


**55 kDa**

**36 kDa**


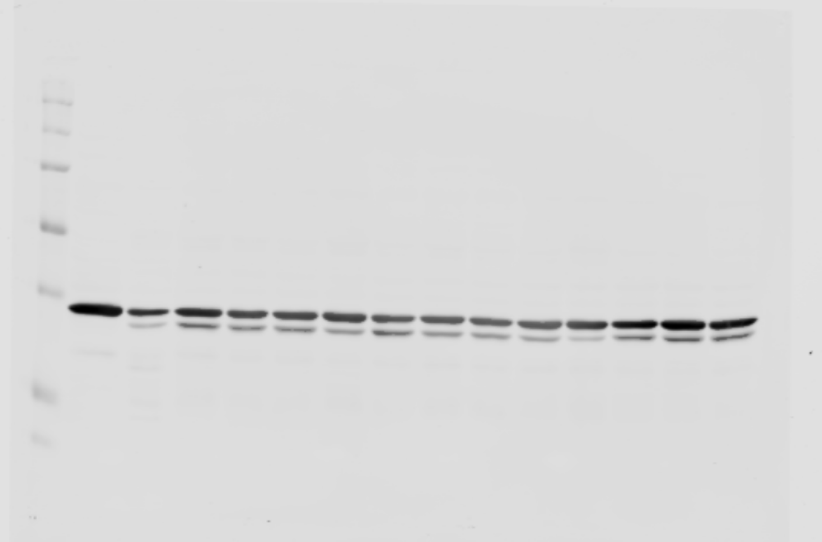

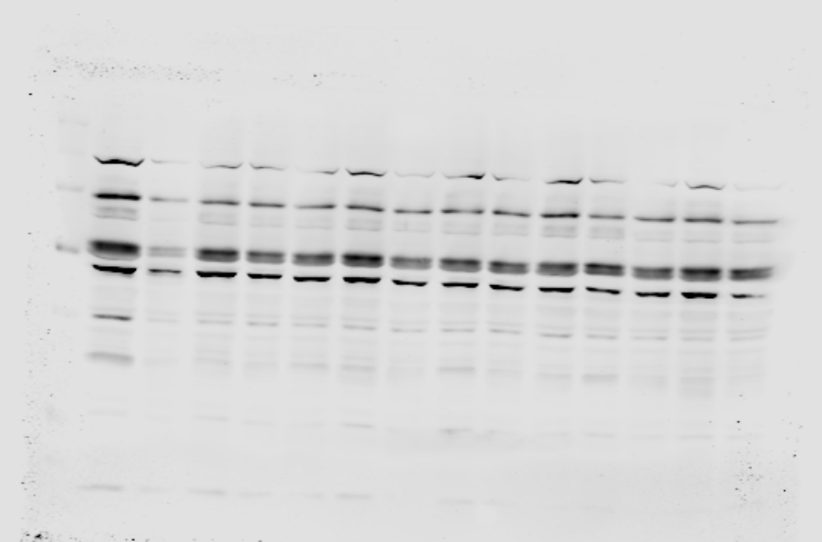


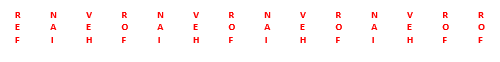


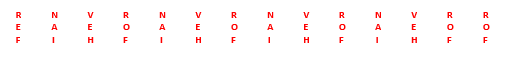


**55 kDa**

**36 kDa**

**Figure S9. Western blot membranes of PKMζ expression in the DH after Test A_1_.** On the left side is the green signal (800 nm) scanning showing PKMζ expression (55 kDa). On the right side is the red signal (680 nm) scan showing GAPDH expression (36 kDa). The sample load in each slot is represented. REF is a hippocampus sample run in all the gels in order to control the scan intensity of the membranes. The red boxes represent the representative cropped bands related to the results expressed in Figure S3B.
